# Biologics for generalized pustular psoriasis: a systematic review and single-arm meta-analysis

**DOI:** 10.3389/fimmu.2024.1462158

**Published:** 2024-10-14

**Authors:** Bai-lin Chen, Qian-wei Liu, Xiao-wan Dong, Yan-ping Bai

**Affiliations:** ^1^ Clinical Medical College, Beijing University of Chinese Medicine, Beijing, China; ^2^ Dermatology Department, National Center for Integrated Traditional Chinese and Western Medicine, China-Japan Friendship Hospital, Beijing, China

**Keywords:** generalized pustular psoriasis, biologics, systematic review, meta-analysis, single-arm

## Abstract

**Introduction:**

Generalized pustular psoriasis (GPP) is a rare and potentially life-threatening auto-inflammatory disease. Currently, there are no consensus-based guidelines or universally accepted treatments. Biologics represent a potential therapeutic option. This study systematically assessed the efficacy and safety of biologics in GPP.

**Methods:**

Relevant studies from three databases were systematically searched until June 28, 2024. Statistical information, including the single-arm proportion rate of the outcomes and 95% confidence intervals (CIs), was analyzed to determine treatment effects. Heterogeneity was assessed using I² values, and subgroup analyses were performed based on drug targets and treatment durations. Data were quantitatively synthesized using a random-effects meta-analysis. Analyses were performed using R statistical software version 4.4.0.

**Results:**

A total of 329 patients from 16 studies were included. The proportion of responders treated with IL-36 inhibitors and IL-17 inhibitors is higher than those treated with TNF-α inhibitors and IL-23 inhibitors. IL-36 inhibitors appear to achieve the highest response rates between 4 and 8 weeks, while IL-17 inhibitors, TNF-alpha inhibitors, and IL-23 inhibitors show a gradual increase in response rates up to 12 weeks. IL-36 inhibitors achieve a 40% (95% CI: 27%-54%) GPPASI75 response rate and a 55% (95% CI: 41%-68%) GPPGA (0,1) response rate within 2 weeks, significantly outperforming other biologics. The recurrence rates of GPP within 52 weeks, ranked from highest to lowest, are: IL-36 inhibitors (21% [95% CI: 9%-28%]), TNF-alpha inhibitors (20% [95% CI: 2%-46%]), IL-17 inhibitors (15% [95% CI: 1%-37%]), and IL-23 inhibitors (5% [95% CI: 0%-29%]). Additionally, 6% (95% CI: 1%-11%) of patients experienced severe adverse events.

**Discussion:**

This meta-analysis highlights the efficacy and safety of biologics in patients with GPP, offering valuable evidence to guide future clinical practice. IL-36 inhibitors show a faster and more substantial clinical response in GPP compared to other biologics. Further research is necessary to assess their role in specific subpopulations and to evaluate their potential long-term effects on flare prevention.

## 1 Introduction

Generalized pustular psoriasis (GPP) is a rare and potentially life-threatening auto-inflammatory disease, characterized by recurrent, sudden flares of widespread painful erythema covered with sterile pustules that may coalesce to form lakes of pus (1–3). Reported mortality rates, ranging from 4% to 24%, underscore the severity of this condition (2, 4, 5).

Conventional treatments such as retinoids, cyclosporine, and methotrexate may be effective in certain cases of GPP, but they often come with significant side effects and may be inadequate for more severe cases (6, 7). With the advent of new biologic therapies, there are now more suitable treatment options available. The joint guidelines from the American Academy of Dermatology and National Psoriasis Foundation recommend infliximab, adalimumab, ustekinumab, secukinumab, ixekizumab, and brodalumab as effective monotherapies for treating GPP in adults (8, 9). Additionally, the US Food and Drug Administration (FDA) approved spesolimab in September 2022 for treating GPP flares in adults, based on data from two trials (10, 11). However, there are currently no consensus-based guidelines or universally accepted treatments available.

To date, there have been no meta-analyses on the use of biologics for GPP, and the available randomized controlled trials (RCTs) (11, 12) are insufficient for such an analysis. The low prevalence and relapsing-remitting nature of GPP make obtaining high-quality evidence on the efficacy and safety of treatments challenging. Additionally, the potential severity of acute flares poses significant challenges for conducting randomized placebo-controlled trials in this population (13). Therefore, we conducted a single-arm meta-analysis to assess the efficacy and safety of biologics in treating GPP.

## 2 Methods

This systematic review and meta-analysis adhered to the Preferred Reporting Items for Systematic Reviews and Meta-Analyses (PRISMA) guidelines (14). The protocol was registered with PROSPERO (CRD42024564157).

### 2.1 Data source and search strategy

Two authors (B.L.C and Q.W.L) conducted an online search on PubMed, Embase, and the Cochrane Central Register of Controlled Trials (CENTRAL) from inception to June 28, 2024. The search keywords included combinations of terms related to biologics, generalized pustular psoriasis, and clinical trials. The complete search strategy is detailed in **Supplementary Table S1**. Additionally, a manual search of the reference lists of screened articles was performed. No language restrictions were applied.

### 2.2 Eligibility criteria and study selection

Eligibility criteria were: (1) RCTs, single-arm trials and observational studies involving patients with GPP; (2) studies involving biologics for GPP; (3) studies reporting Generalized Pustular Psoriasis Area and Severity Index (GPPASI), Generalized Pustular Psoriasis Physician Global Assessment (GPPGA), the GPP flare during maintenance, or adverse events (15–17). Titles and abstracts of potential studies were independently screened by two authors (B.L.C and Q.W.L). Irrelevant studies were excluded based on the following criteria: (1) duplicate studies from the same trials; (2) reviews and case reports. Full texts were assessed for eligibility when abstracts provided insufficient information. Discrepancies were resolved through discussion with the senior author (Y.P.B).

### 2.3 Data extraction and evaluation of the risk of bias

The characteristics of included studies were extracted, including authors, registration number, study period, funding sources, race of participants, region, sample size, age, sex, treatment regimens, and outcomes such as the proportion of GPPASI 75/90/100, GPPGA (0, 1), GPP flare during maintenance, and adverse events (15–17). Two authors (B.L.C and Q.W.L) independently assessed the quality using the Risk Of Bias In Non-randomized Studies - of Interventions (ROBINS-I) tool (18), the Cochrane Collaboration risk of bias tool (19), and the GRADE assessment (20). Consistency was assessed using the kappa coefficient, as shown in **Table 1**. Any disagreements were resolved through consensus with the senior author (Y.P.B).

**Table 1:** Demographic data.

GPP (n=329)		
**Sex (n=329)**	**Female**	179 (54.4%)
	**Male**	150 (45.6%)
**Weight, kg (n=226)**		52.0 (12.5)1
**BMI, kg/m2 (n=163)**		26.0 (7.2)1
**Age,y (n=283)**		46.6 (16.65)1
**Race (n=329)**	**White**	98 (29.8%)
	**Asian**	230 (69.9)1
	**N/A**	1 (0.3%)
**Baseline GPPASI (n=159)**		11.2 (14.6)1
**IL36RN mutation (n=166)**		37 (22.3%)
**CARD14 mutation (n=67)**		7 (10.4%)
**AP1S3 mutation (n=50)**		1 (2%)

^1^Reported as mean (SD). Bold values represent the total number of study subjects for which relevant information is available.

### 2.4 Data synthesis and statistical analysis

Statistical information, including the single-arm proportion rate of the outcomes and 95% confidence intervals (CIs), was analyzed to determine treatment effects. Heterogeneity was assessed using I² values, and subgroup analyses were performed based on drug targets and treatment durations. Data were quantitatively synthesized using a random-effects meta-analysis. All tests were two-sided, and a p-value < 0.05 was considered significant. Funnel plots and the Egger test were used to evaluate publication bias in the included studies. Sensitivity analyses were conducted to assess the robustness and reliability of the combined results. Analyses were performed using R statistical software version 4.4.0.

## 3 Results

### 3.1 Study characteristics

**Figure 1:**
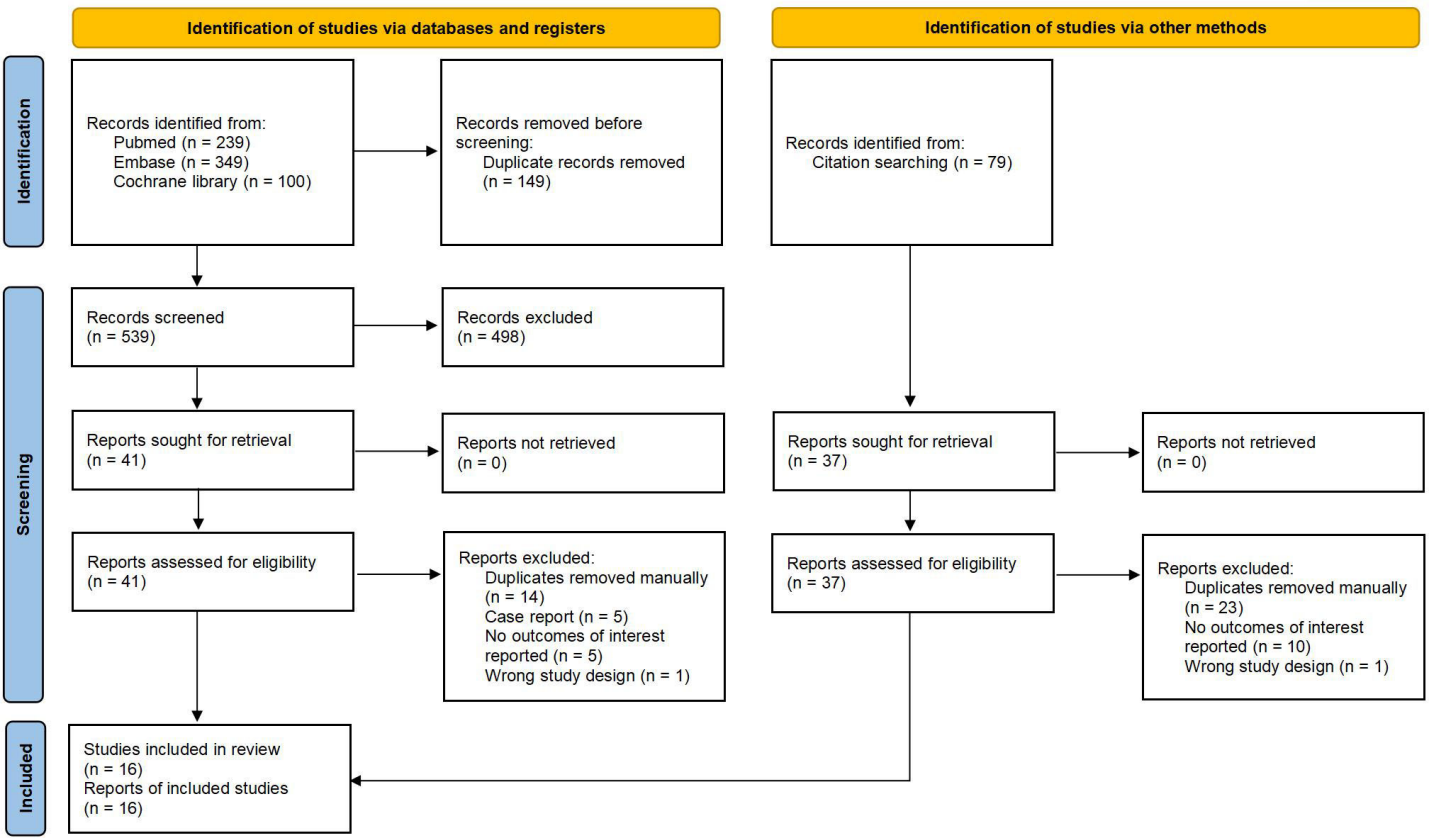
Study flow diagram.

Out of the 767 articles identified, 16 trials met the inclusion criteria (**Figure 1**). The demographic data and characteristics of the included studies are summarized in **Table 1**, **2** (10–12, 21–33). The excluded trials are listed in **Supplementary Table S3**. One trial was funded by a not-for-profit foundation (23). One trial was with no funding support (21). And the remaining trials received funding from the pharmaceutical industry (10–12, 22, 24–33). Four trials were conducted in multiple countries (10–12, 31), while the rest were conducted in Italy (21), China (23) or Japan (22, 24–30, 32, 33). The total sample size consisted of 329 patients: 157 patients received IL-36 targeted biologics (spesolimab, imsidolimab); 97 patients received IL-17 targeted biologics (brodalumab, ixekizumab, secukinumab); 43 patients received IL-23 targeted biologics (guselkumab, risankizumab); 32 patients received TNF-α targeted biologics (adalimumab, certolizumab).

**Table 2:** Characteristics of included studies.

Study	Study design	Drug	Target	Duration	Region	Race	No. of participants	Age, y	Female, %	Weight, kg	BMI, kg/m^2^	Outcomes measures	Baseline GPPASI	Gene mutation
Avallone 2023 (21)	Retrospective cohort study	NR	IL-23 IL-17	NR	Italy	White: 36	36	IL-23: 32.0 (20.0-48.0)^1^ IL-17: 39.0 (20.0-52.0)^1^	58.3	IL-23: 67.0 (58.0-78.5)^1^ IL-17: 79.0 (70.0-86.0)^1^	NR	GPPASI, GPPGA, AE	NR	NR
Bachelez 2019 (10)	Prospective single-arm trial	Spesolimab	IL-36	Once	Tunisia, France, Malaysia, Korea, Taiwan	Asian: 4 White: 2 N/A: 1	7	38.6 (13.8)^2^	57.1	62.8 (11.0)^2^	23.3 (4.3)^2^	GPPASI, GPPGA, C-reactive protein (CRP), AE	27.5 (12.3)^2^	IL36RN: 3 CARD14: 1
Bachelez 2021 (11)	Randomized controlled trial	Spesolimab	IL-36	Once	China, France, Germany, Japan, Korea, Malaysia, Singapore, Switzerland, Taiwan, Thailand, Tunisia, United States	White: 23 Asian: 27	50	43.0 (11.0)^2^	72.0	73.7 (24.0)^2^	NR	GPPASI, GPPGA, Pain on a visual analogue scale (pain VAS), Psoriasis Symptom Scale (PSS), Functional Assessment of Chronic Illness Therapy–Fatigue (FACIT-Fatigue), AE	NR	IL36RN: 7 CARD14: 5 AP1S3: 1
Imafuku 2016 (22)	Prospective single-arm trial	Secukinumab	IL-17	52 w	Japan	Asian: 12	12	66.7 (15.3)^2^	66.7	70.7 (16.3)^2^	26.16 (5.84)^2^	CGI, Japanese Dermatological Association (JDA) score, GPPASI, AE	NR	NR
Lu 2024 (23)	Prospective cohort study	Adalimumab Guselkumab Secukinumab	TNF-α IL-23 IL-17	12 w	China	Asian: 50	50	49.9 (15.6)^2^	26.0	NR	TNF-α: 24.7 (23.2- 27.6)^1^ IL-23: 23.3 (21.8-24.6)^1^ IL-17: 25.4 (23.1-28.2)^1^	GPPASI, GPPGA, Body Surface Area (BSA), Dermatology Life Quality Index (DLQI), AE	NR	NR
Morita 2018 (24)	Prospective single-arm trial	Adalimumab	TNF-α	52 w	Japan	Asian: 10	10	49.8 (13.3)^2^	30.0	70.7 (12.5)^2^	NR	The total skin score, systemic/laboratory score, Physician Global Assessment (PGA), GPPASI, DLQI, 36-Item Short Form Health Survey (SF-36), AE	28.3 (16.0)^2^	NR
Morita 2022 (25)	Prospective single-arm trial	Ixekizumab	IL-17	12 w	Japan	Asian: 7	7	45.0 (19.1)^2^	57.1	67.9 (20.2)^2^	25.9 (5.73)^2^	Global improvement score (GIS), GPPASI, GPPGA, PSS, BSA, AE	10.2	IL36RN: 1
Morita 2023 (12)	Randomized controlled trial	Spesolimab	IL-36	48 w	Argentina, Belgium, Chile, China, France, Germany, Italy, Japan, Malaysia, Mexico, Philippines, Republic of Korea, Russia, Spain, Taiwan, Thailand, Tunisia, Turkey, USA, Vietnam	White = 30 Asian = 62	92	40.7 (16.5)^2^	63.0	26.7 (7.8)^2^	26.7 (7.9)^2^	PSS, DLQI, AE	3.4 (4.1)^2^	IL36RN: 24
Nagata 2020 (26)	Case series	Ixekizumab	IL-17	NR	Japan	Asian: 10	10	65.2 (47.0-89.0)^3^	30.0	NR	NR	GPPASI, AE	25.5 (9.5)^2^	IL36RN: 2 CARD14: 1
Okubo 2019 (27)	Prospective single-arm trial	Ixekizumab	IL-17	52 w	Japan	Asian: 5	5	47.8 (21.36)^2^	60.0	56.0 (9.0)^2^	NR	GIS, GPPASI, DLQI, Itch Numeric Rating Scale (INRS), AE	12.8 (5.5)^2^	NR
Okubo 2022 (28)	Prospective single-arm trial	Certolizumab	TNF-α	52 w	Japan	Asian: 7	7	48.3 (12.5)^2^	57.1	60.1 (14.5)^2^	23.3 (6.7)^2^	CGI, DLQI, INRS, GIS, JDA score, GPPASI, PGA, AE	NR	NR
Saeki 2015 (29)	Prospective single-arm trial	Ixekizumab	IL-17	52 w	Japan	Asian: 5	5	48.2 (15.6)^2^	60.0	55.8 (10.2)^2^	NR	GPPASI, GPPGA, INRS, GIS, NAPSI, PSSI, DLQI, AE	12.8 (5.5)^2^	NR
Sano 2018 (30)	Prospective single-arm trial	Guselkumab	IL-23	52 w	Japan	Asian: 10	10	42.6 (8.97)^2^	40.0	NR	26.9 (6.39)^2^	CGI, JDA score, GPPASI, Investigator’s Global Assessment (IGA), DLQI, Physical Component Scores (PCS), SF-36, AE	29.3 (20.0)^2^	NR
Warren 2023 (31)	Prospective single-arm trial	Imsidolimab	IL-36	12 w	UK, Poland	White = 7 Asian = 1	8	51.3 (14.9)^2^	50.0	78.8 (13.3)^2^	28.9 (3.4)^2^	CGI, BSA, GPPPGA, DLQI, AE	NR	NR
Yamasaki 2017 (32)	Prospective single-arm trial	Brodalumab	IL-17	52 w	Japan	Asian: 12	12	43.1 (16.8)^2^	75.0	58.6 (16.7)^2^	22.5 (5.5)^2^	CGI, GPPASI, sPGA, BSA, PSS, NAPSI, DLQI, Psoriasis Disability Index (PDI), SF‐36, AE	15.0 (12.1)^2^	NR
Yamanaka 2023 (33)	Prospective single-arm trial	Risankizumab	IL-23	160 w	Japan	Asian: 8	8	57.5 (18.7)^2^	25.0	68.8 (2.9)^2^	23.9 (4.2)^2^	JDA score, GPPASI, DLQI, AE	17.4 (9.4)^2^	NR

^1^ Reported as median (IQR).

^2^ Reported as mean (SD).

^3^ Reported as median (Range).

^4^ Reported as mean.

### 3.2 Risk of bias assessment

ROBINS-I is a new tool used for evaluating the risk of bias in observational studies (18). Cochrane Collaboration risk of bias tool (19) was utilized for assessing the risk of bias in randomized controlled trials. Detailed information can be found in **Table 3**.

**Table 3:** Risk of Bias Assessment.

Study	Type of bias							Overall rating
	Bias due to confounding	Bias due to selection of participants	Bias due to exposure assessment	Bias due to misclassification during follow-up	Bias due to missing data	Bias due to measurement of the outcome	Bias due to selective reporting of the results	
Avallone 2023 (21)	Moderate	Low	Low	Low	Low	Moderate	Low	Moderate
Bachelez 2019 (10)	Low	Low	Low	Low	Low	Moderate	Low	Moderate
Imafuku 2016 (22)	Serious	Serious	Low	Serious	Low	Moderate	Low	High
Lu 2024 (23)	Low	Low	Low	Low	Low	Moderate	Low	Moderate
Morita 2018 (24)	Serious	Moderate	Low	Serious	Low	Moderate	Low	High
Morita 2022 (25)	Low	Moderate	Low	Low	Low	Moderate	Low	Moderate
Nagata 2020 (26)	Serious	Moderate	Low	Serious	Low	Moderate	Low	High
Okubo 2019 (27)	Low	Low	Low	Low	Low	Moderate	Low	Moderate
Okubo 2022 (28)	Serious	Serious	Low	Low	Low	Moderate	Low	High
Saeki 2015 (29)	Low	Low	Low	Low	Low	Moderate	Low	Moderate
Sano 2018 (30)	Serious	Low	Low	Serious	Low	Moderate	Low	High
Warren 2023 (31)	Low	Low	Low	Low	Low	Moderate	Low	Moderate
Yamasaki 2017 (32)	Moderate	Low	Low	Moderate	Low	Moderate	Low	Moderate
Yamanaka 2023 (33)	Moderate	Low	Low	Serious	Low	Moderate	Low	Moderate
Kappa	0.70	0.91	1.00	1.00	1.00	1.00	1.00	0.86
Study	Type of bias							Overall rating
	Random sequence generation	Allocation concealment	Blinding of participants and personnel	Blinding of outcome assessment	Incomplete outcome data addressed	Selective reporting		
Bachelez 2021 (11)	Low	Low	Low	NI	Low	Low		Low
Morita 2023 (12)	Low	Low	Low	Low	Low	Low		Low
Kappa	1.00	1.00	1.00	1.00	1.00	1.00		1.00

### 3.3 GPPASI

**Figure 2:**
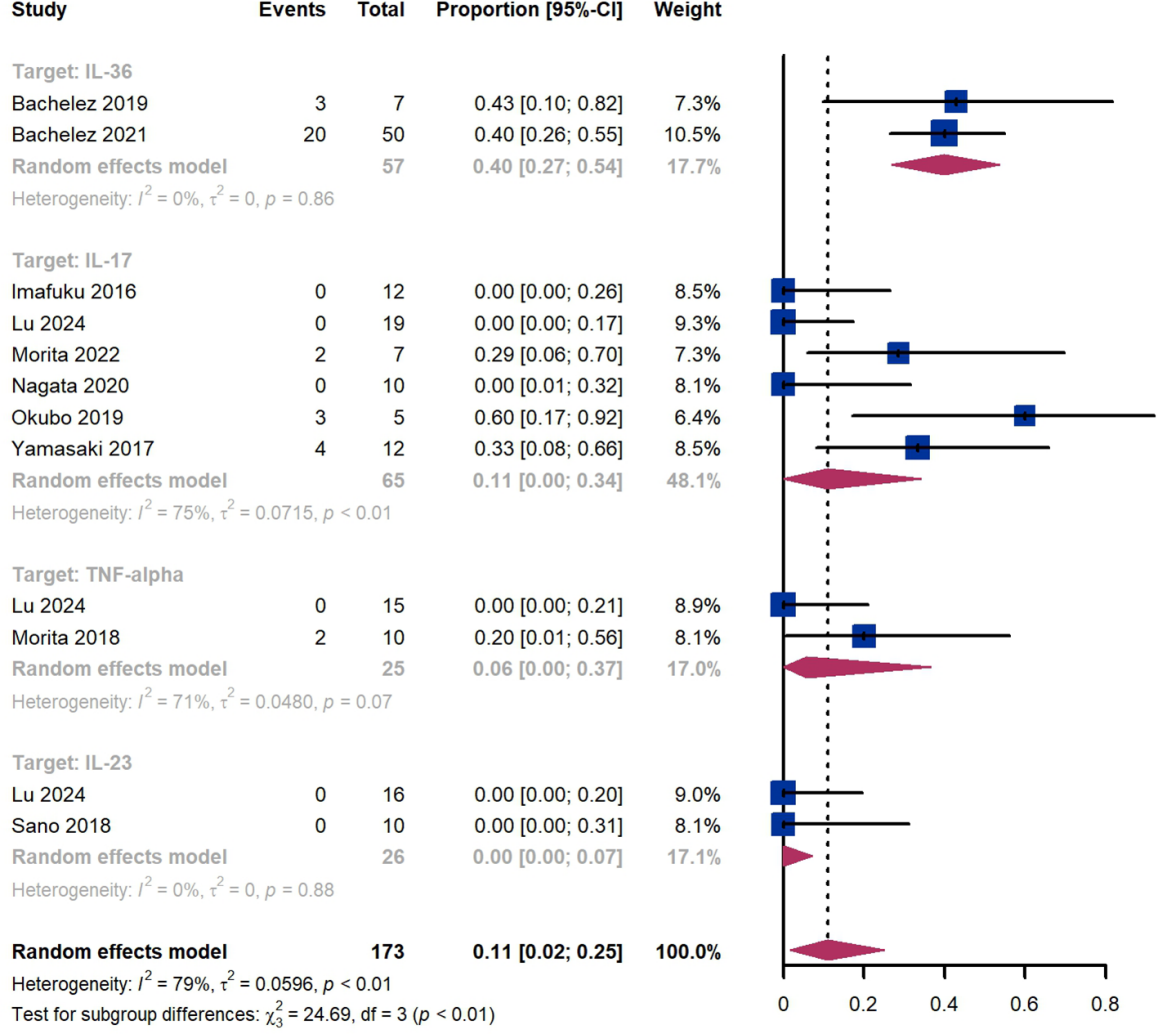
Pooled analysis of responders’ proportion for GPPASI 75 (2 w).

Within 2 weeks, we observed a GPPASI 75 responder proportion of 11% (95% CI 2%-25%) among 173 patients, including 40% (95% CI 27%-54%) for patients treated by IL-36 targeted biologics, 11% (95% CI 0%-34%) for those treated by IL-17 targeted biologics, 6% (95% CI 0%-37%) for those treated by TNF-α targeted biologics, 0% (95% CI 0%-7%) for those treated by IL-23 targeted biologics (**Figure 2**). The Egger test for publication bias indicated evidence of bias (P = 0.52).

**Figure 3:**
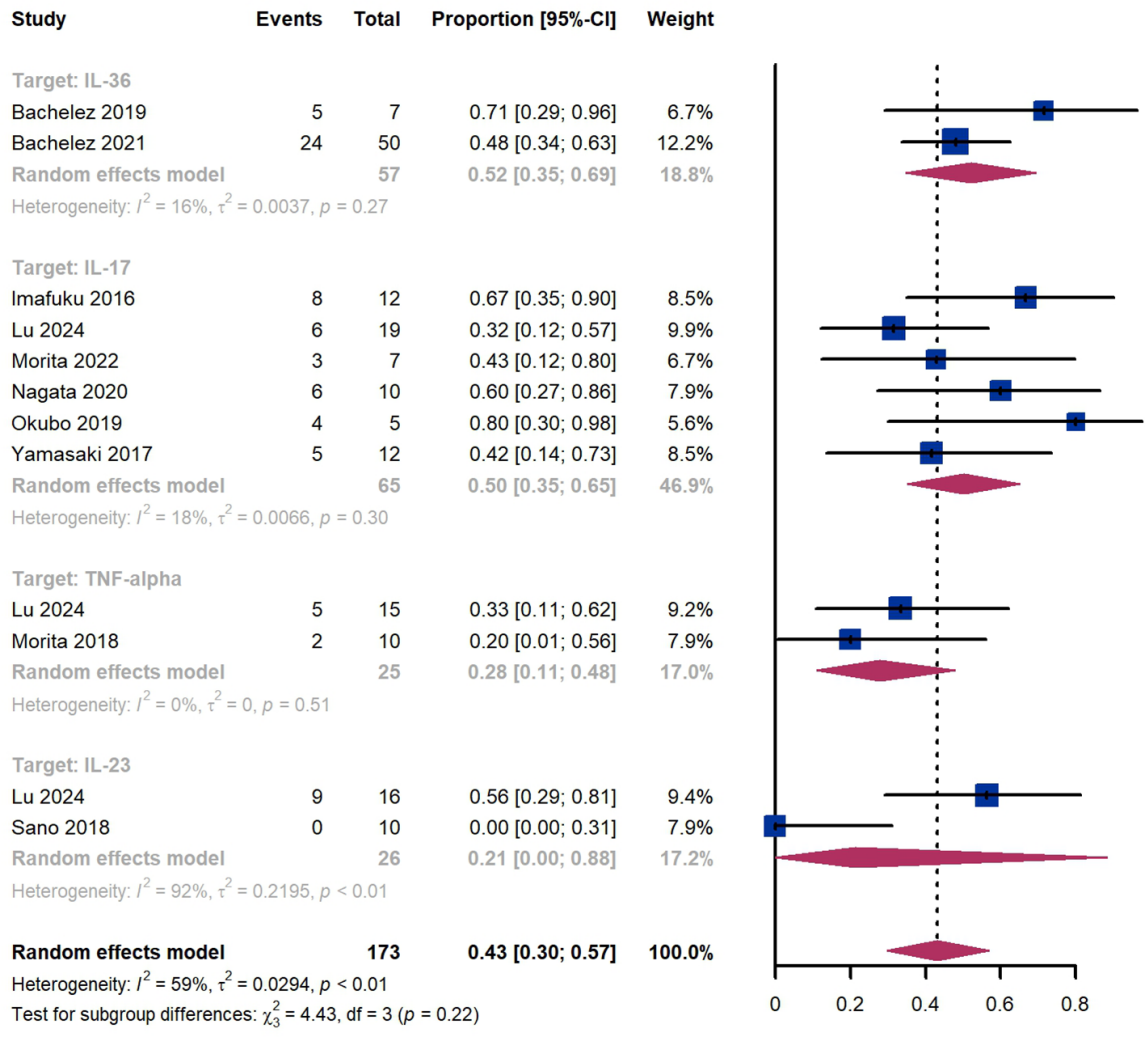
Pooled analysis of responders’ proportion for GPPASI 75 (4 w).

Within 4 weeks, we observed a GPPASI 75 responder proportion of 43% (95% CI 30%-57%) among 173 patients, including 52% (95% CI 35%-69%) for patients treated by IL-36 targeted biologics, 50% (95% CI 35%-65%) for those treated by IL-17 targeted biologics, 28% (95% CI 11%-48%) for those treated by TNF-α targeted biologics (**Figure 3**). The Egger test for publication bias indicated evidence of bias (P = 0.97).

**Figure 4:**
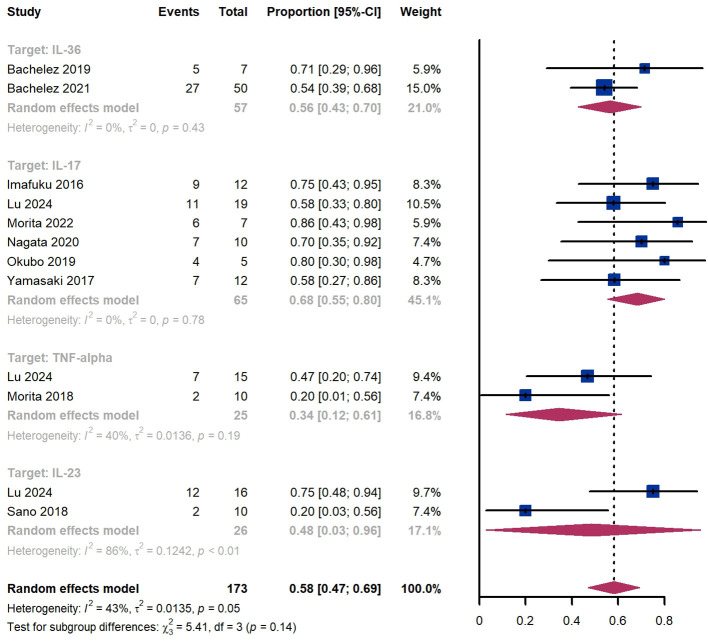
Pooled analysis of responders’ proportion for GPPASI 75 (8 w).

Within 8 weeks, we observed a GPPASI 75 responder proportion of 58% (95% CI 47%-69%) among 173 patients, including 56% (95% CI 43%-70%) for patients treated by IL-36 targeted biologics, 68% (95% CI 55%-80%) for those treated by IL-17 targeted biologics, 34% (95% CI 12%-61%) for those treated by TNF-α targeted biologics (**Figure 4**). The Egger test for publication bias indicated evidence of bias (P = 0.61).

**Figure 5:**
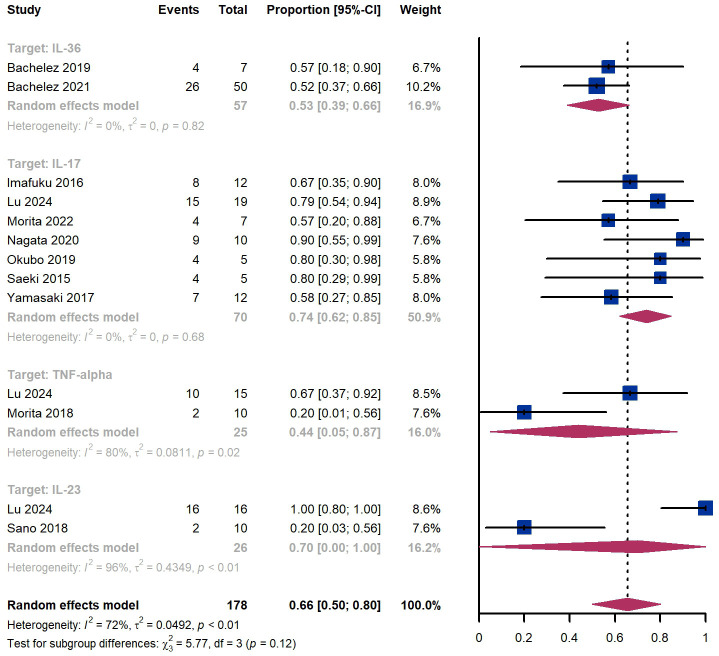
Pooled analysis of responders’ proportion for GPPASI 75 (12 w).

Within 12 weeks, we observed a GPPASI 75 responder proportion of 66% (95% CI 50%-80%) among 178 patients, including 53% (95% CI 39%-66%) for patients treated by IL-36 targeted biologics, 74% (95% CI 62%-85%) for those treated by IL-17 targeted biologics (**Figure 5**). The Egger test for publication bias indicated no evidence of bias (P = 0.79).

And we observed a GPPASI 90 responder proportion of 1% (95% CI 0%-4%) among 123 patients within 2 weeks, 16% (95% CI 7%-28%) among 123 patients within 4 weeks, 36% (95% CI 23%-50%) among 123 patients within 8 weeks, 50% (95% CI 34%-67%) among 172 patients within 12 weeks. As for GPPASI 100, we observed a responder proportion of 0% (95% CI 0%-2%) among 123 patients within 2 weeks, 1% (95% CI 0%-5%) among 123 patients within 4 weeks, 4% (95% CI 0%-12%) among 123 patients within 8 weeks, 9% (95% CI 1%-20%) among 164 patients within 12 weeks.

### 3.4 GPPGA (0, 1)

**Figure 6:**
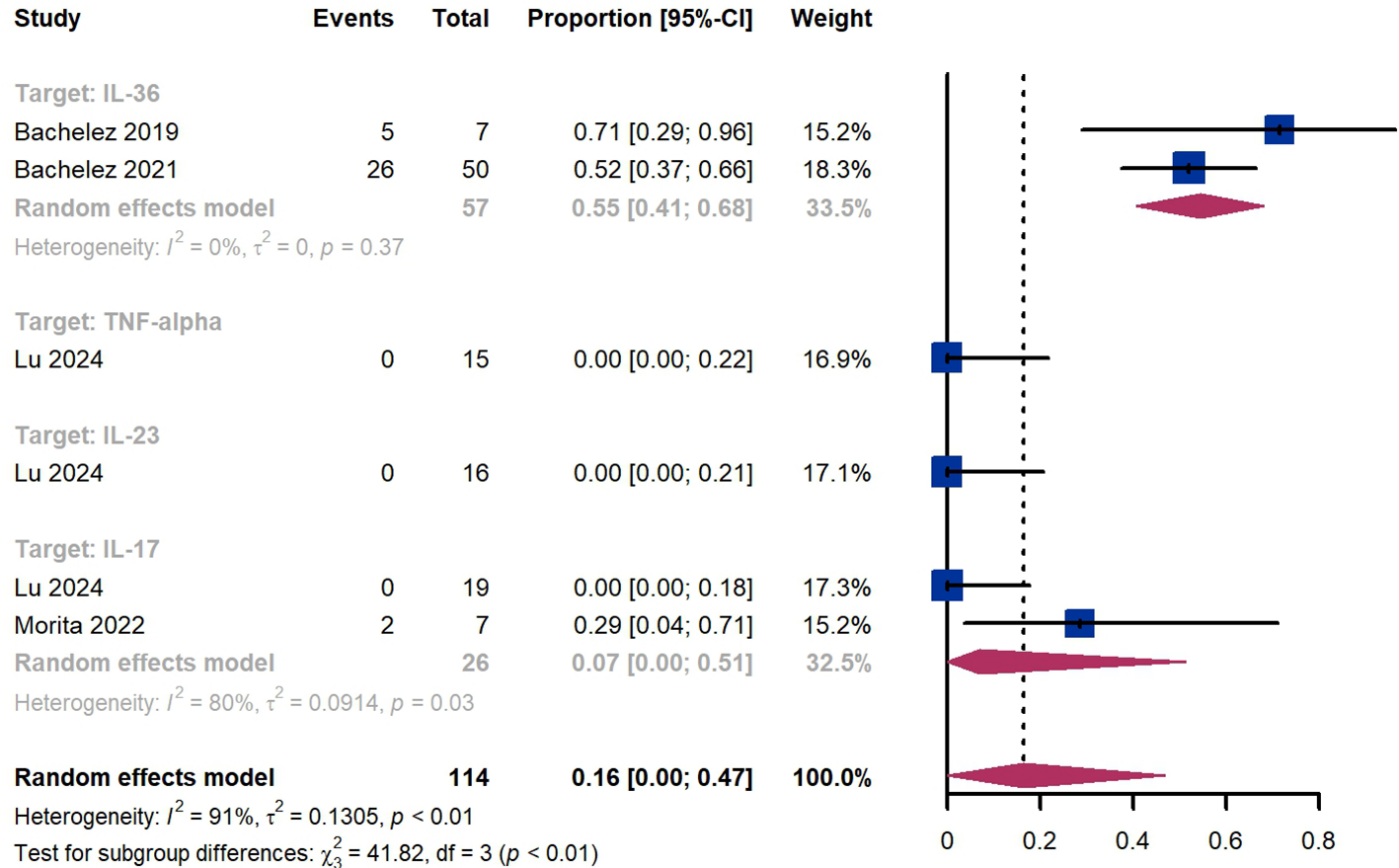
Pooled analysis of responders’ proportion for GPPGA (0, 1) (2 w).

Treatment success was defined as achieving a GPPGA score of 0 or 1. Within 2 weeks, we observed a responder proportion of 16% (95% CI 0%-47%) among 114 patients, including 55% (95% CI 41%-68%) for patients treated by IL-36 targeted biologics, 0% (95% CI 0%-22%) for those treated by TNF-α targeted biologics, 0% (95% CI 0%-21%) for those treated by IL-23 targeted biologics, 7% (95% CI 0-51%) for those treated by IL-17 targeted biologics (**Figure 6**). The Egger test for publication bias indicated no evidence of bias (P = 0.52).

**Figure 7:**
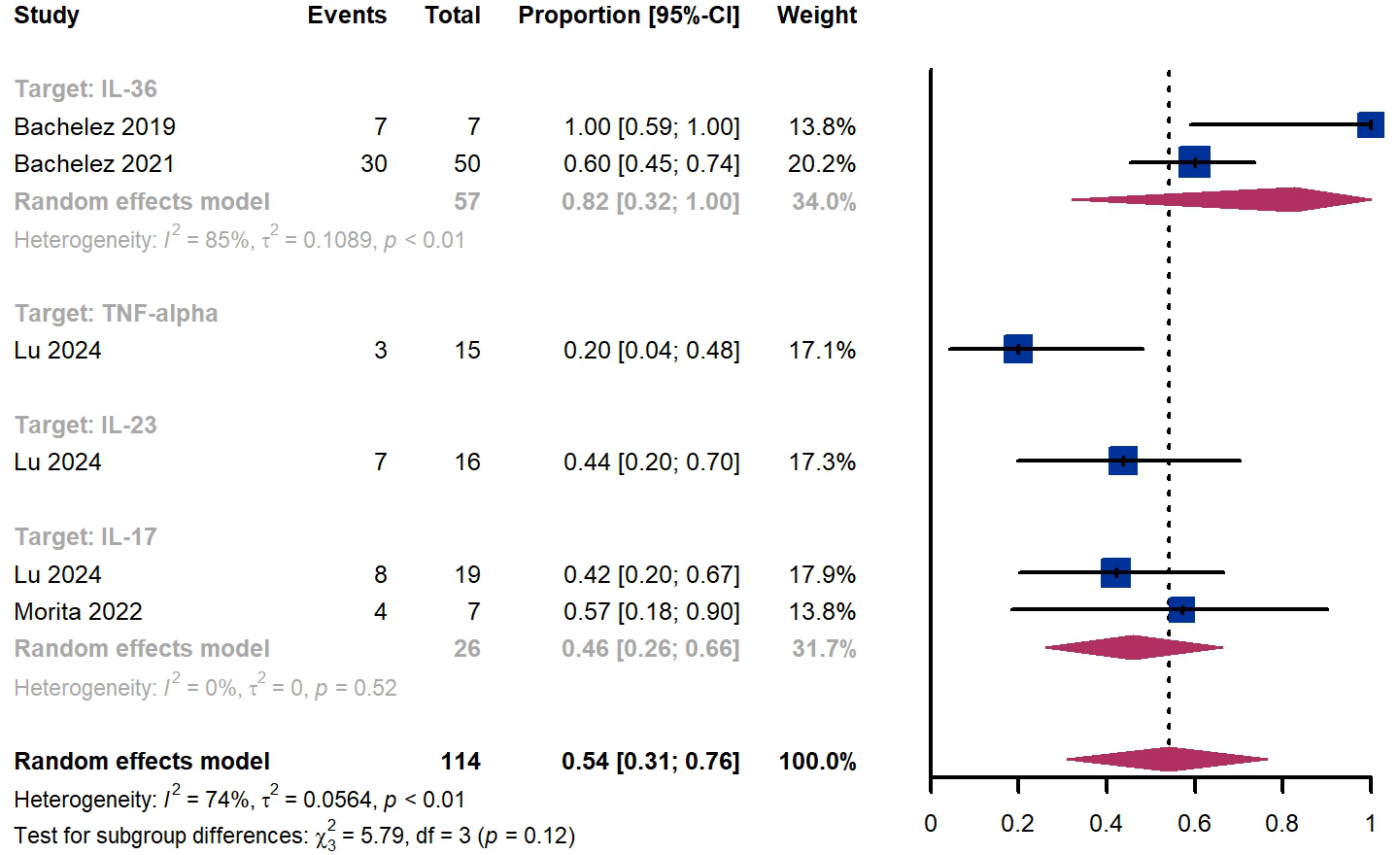
Pooled analysis of responders’ proportion for GPPGA (0, 1) (4 w).

Within 4 weeks, we observed a responder proportion of 54% (95% CI 31%-76%) among 114 patients, including 82% (95% CI 32%-100%) for patients treated by IL-36 targeted biologics, 20% (95% CI 4%-48%) for patients treated by TNF-α targeted biologics, 44% (95% CI 20%-70%) for those treated by IL-23 targeted biologics, 46% (95% CI 26-66%) for those treated by IL-17 targeted biologics (**Figure 7**). The Egger test for publication bias indicated no evidence of bias (P = 0.87).

**Figure 8:**
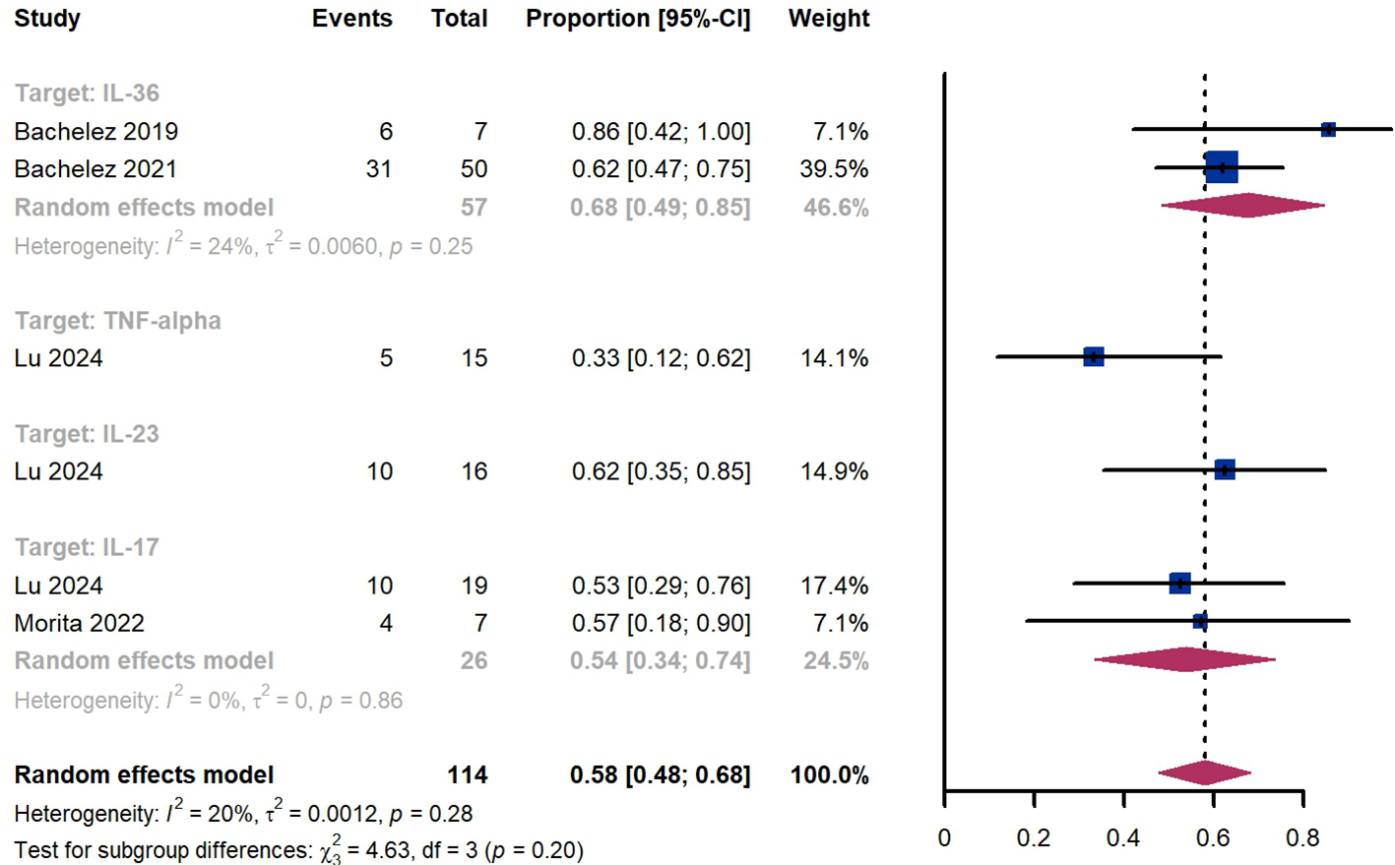
Pooled analysis of responders’ proportion for GPPGA (0, 1) (8 w).

Within 8 weeks, we observed a responder proportion of 58% (95% CI 48%-68%) among 114 patients, including 68% (95% CI 49%-85%) for patients treated by IL-36 targeted biologics, 33% (95% CI 12%-62%) for patients treated by TNF-α targeted biologics, 62% (95% CI 35%-85%) for those treated by IL-23 targeted biologics, 54% (95% CI 34-74%) for those treated by IL-17 targeted biologics (**Figure 8**). The Egger test for publication bias indicated no evidence of bias (P = 0.99).

**Figure 9:**
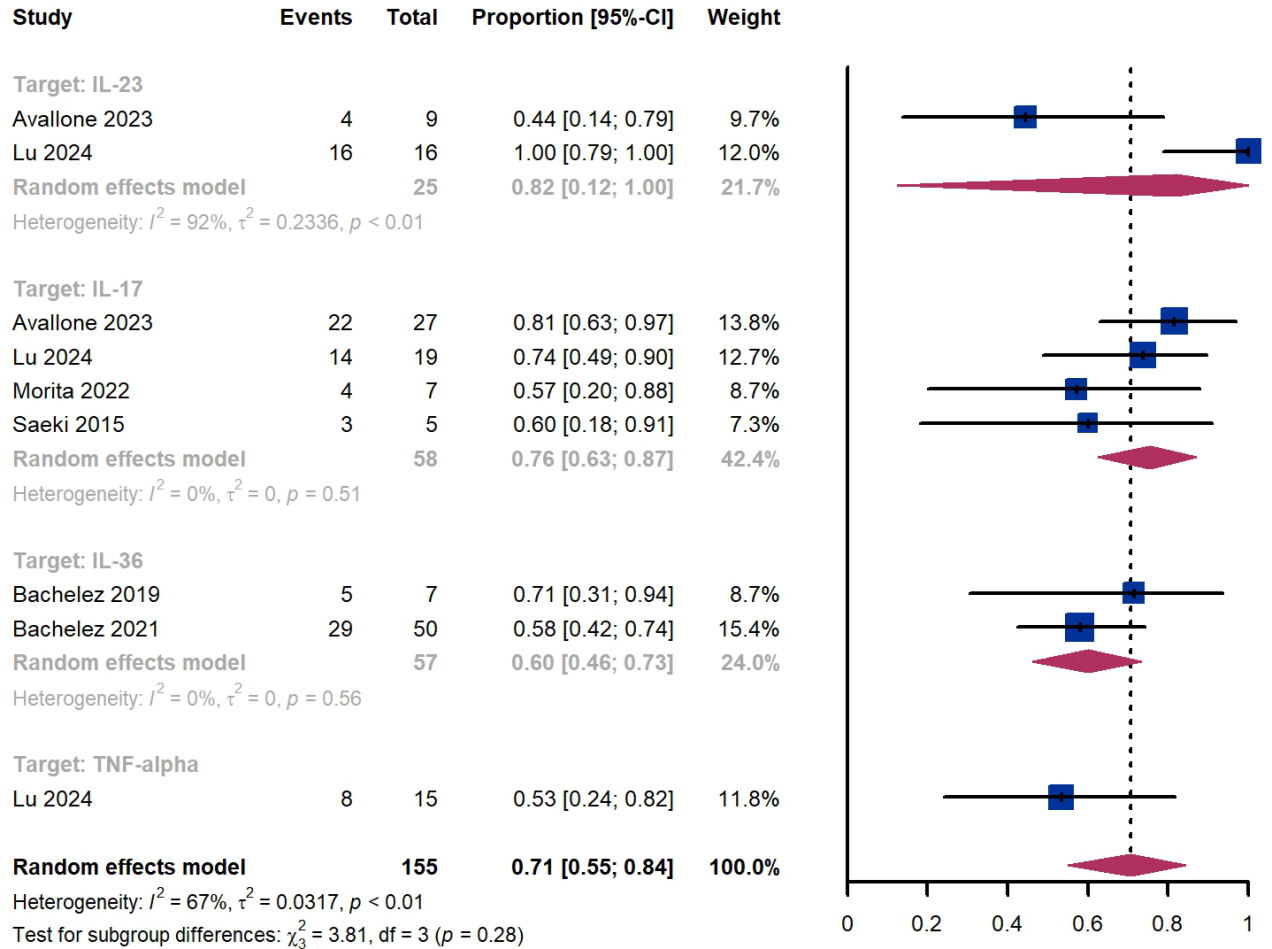
Pooled analysis of responders’ proportion for GPPGA (0, 1) (12 w).

Within 12 weeks, we observed a responder proportion of 71% (95% CI 55%-84%) among 155 patients, including 76% (95% CI 63-87%) for those treated by IL-17 targeted biologics, 60% (95% CI 46%-73%) for patients treated by IL-36 targeted biologics, 53% (95% CI 24%-82%) for those treated by TNF-α targeted biologics, (**Figure 9**). The Egger test for publication bias indicated no evidence of bias (P = 0.97).

### 3.5 GPP flare

**Figure 10:**
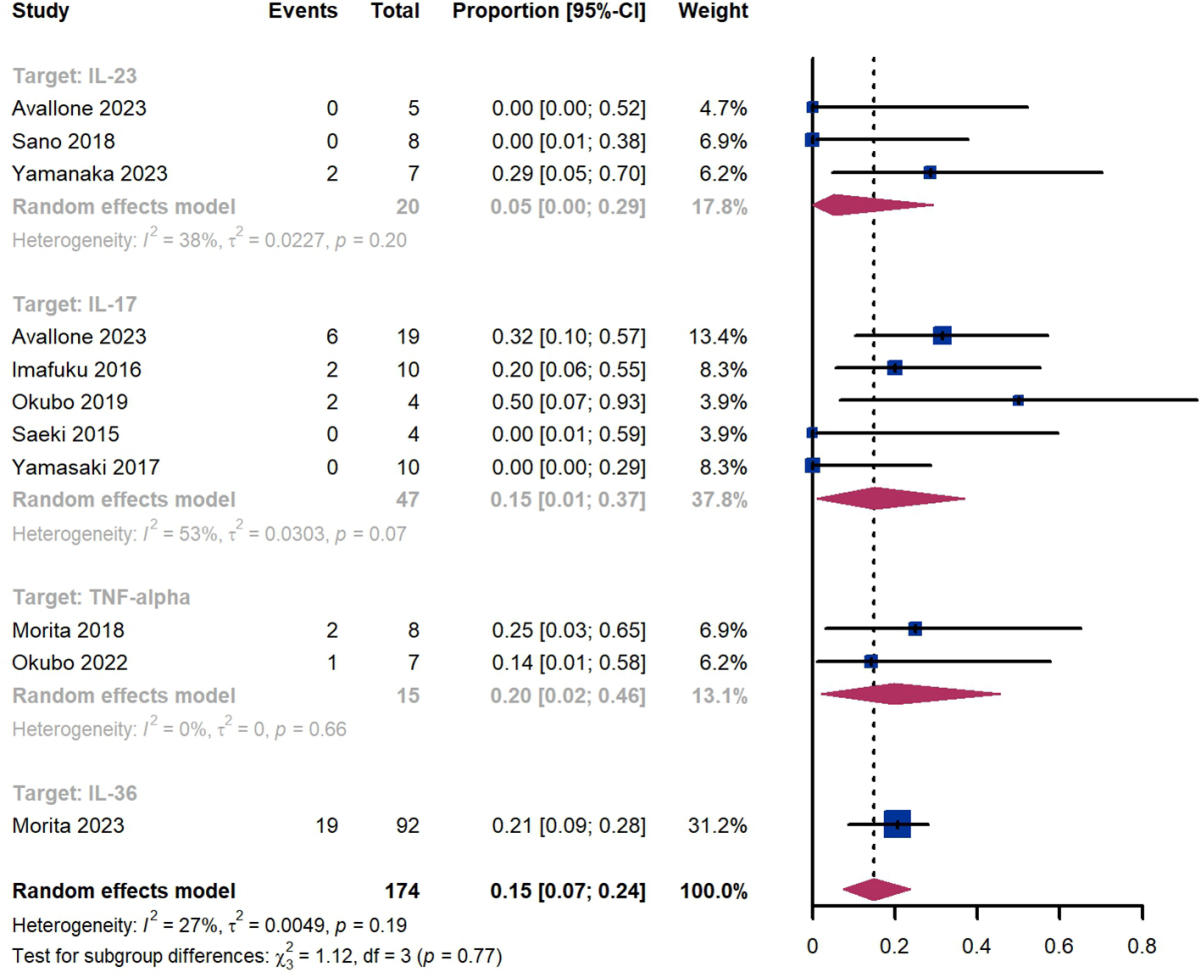
Pooled analysis of proportion for flares within 52 weeks.

Within 52 weeks, we observed a GPP flare proportion of 15% (95% CI 7%-24%) among 174 patients, including 5% (95% CI 0%-29%) for those treated by IL-23 targeted biologics, 15% (95% CI 1-37%) for those treated by IL-17 targeted biologics, 20% (95% CI 2%-46%) for those treated by TNF-α targeted biologics, 21% (95% CI 9%-28%) for patients treated by IL-36 targeted biologics (**Figure 10**). The Egger test for publication bias indicated no evidence of bias (P = 0.52).

Within 52 weeks, we observed a GPP flare proportion of 15% (95% CI 7%-24%) among 174 patients, including 5% (95% CI 0%-29%) for those treated by IL-23 targeted biologics, 15% (95% CI 1-37%) for those treated by IL-17 targeted biologics, 20% (95% CI 2%-46%) for those treated by TNF-α targeted biologics, 21% (95% CI 9%-28%) for patients treated by IL-36 targeted biologics (**Figure 10**). The Egger test for publication bias indicated no evidence of bias (P = 0.52).

### 3.6 Adverse events

**Figure 11:**
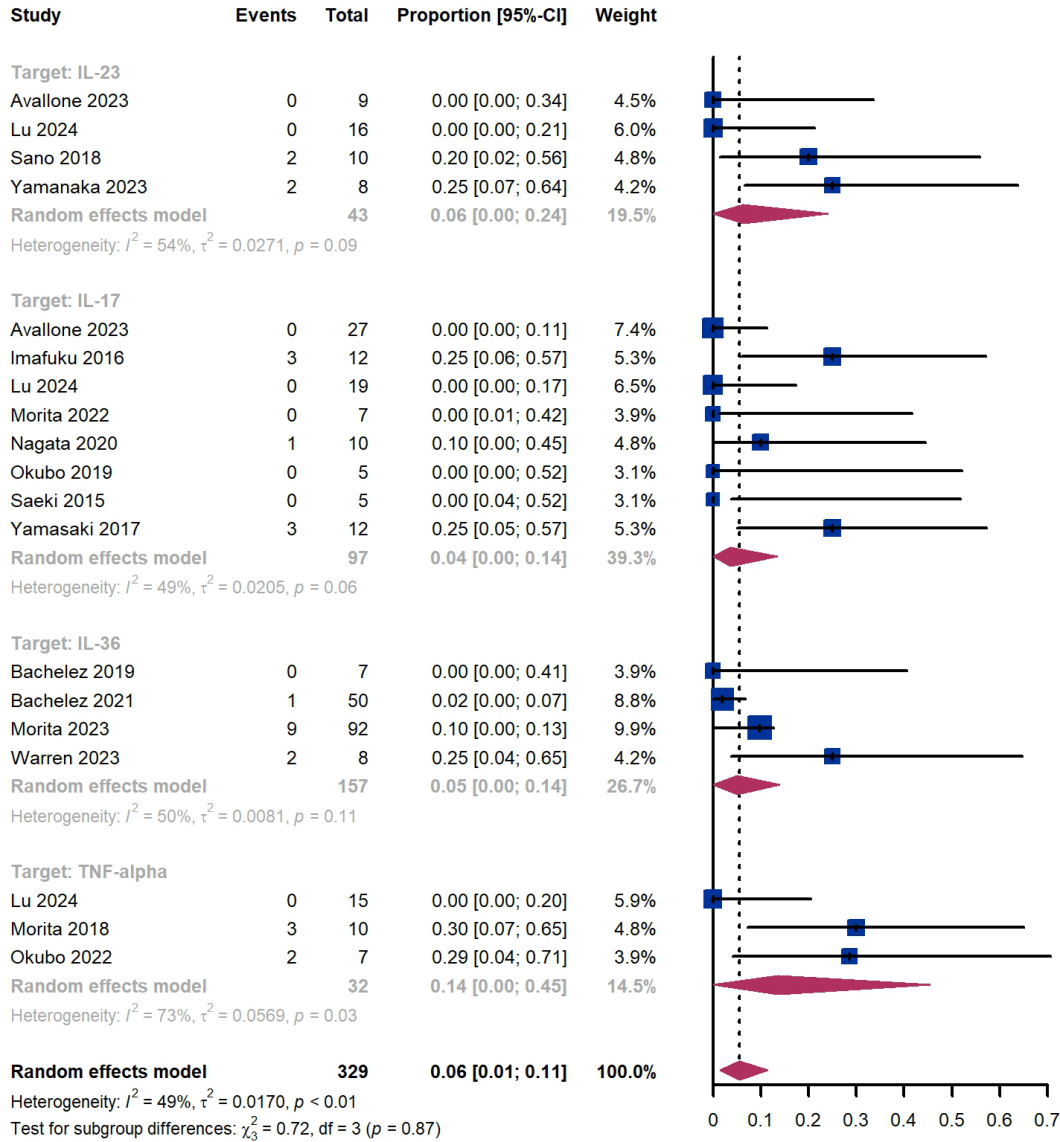
Pooled analysis of patients’ proportion for severe adverse events.

Out of 329 patients, 249 experienced adverse events, resulting in an incidence rate of 75.7%. The most common side effects included infection (20.4%), skin and subcutaneous tissue disorders (16.1%), injection site reaction (12.5%), and dry mucosa (12.2%) (**Supplementary Table S4**). Additionally, we observed that 6% (95% CI 1%-11%) of patients experienced severe adverse events, including 6% (95% CI 0%-24%) for those treated by IL-23 targeted biologics, 4% (95% CI 0-14%) for those treated by IL-17 targeted biologics, 5% (95% CI 0-14%) for patients treated by IL-36 targeted biologics, 14% (95% CI 0%-45%) for those treated by TNF-α targeted biologics (**Figure 11**). The Egger test for publication bias indicated no evidence of bias (P = 0.29). A funnel plot is provided in **Supplementary Figure S1**.

### 3.7 Sensitivity analysis

We evaluated the impact of each study on the pooled results for the proportion of all outcomes to demonstrate stability and sensitivity (**Supplementary Figures S2**). With the exception of minor adjustments in CIs, the overall incidences obtained through the combined analysis remained consistent across all included studies. This suggests that our estimated proportion of responders is relatively robust and conservative.

## 4 Discussion

This systematic review examines the effectiveness and safety of biologics in treating GPP. Dysregulation of the IL-36 inflammatory pathway appears to be the main driver of GPP pathogenesis (34). IL-23 regulates the synthesis of IL-17, which in turn stimulates the production of pro-inflammatory IL-36R agonists, further over-activating the IL-36 pathway (35). TNF-α is associated with increased production of IL-36R agonists, which stimulate the IL-36 pathway and induce more TNF-α production in a continuous inflammatory loop. TNF-α inhibitors indirectly suppress the expression of IL-36γ, thereby reducing activation of the pro-inflammatory IL-36 pathway (36, 37).

This study indicates that the proportion of responders achieving GPPASI 75 and GPPGA (0, 1) tends to increase over the course of 12 weeks. Subgroup analysis by targets shows that responders treated with IL-36 and IL-17 inhibitors have higher rates than those treated with TNF-α and IL-23 inhibitors. IL-36 inhibitors demonstrates better efficacy than IL-17 inhibitors at 2 and 4 weeks, whereas IL-17 inhibitors surpass the efficacy of IL-36 inhibitors at 8 and 12 weeks. IL-36 inhibitors seem to reach the highest response rates between 4 and 8 weeks, while IL-17, TNF-alpha, and IL-23 inhibitors show a gradual increase in response rates up to 12 weeks.

GPP is characterized by recurrent, sudden flares of widespread painful erythema covered with sterile pustules, which may coalesce into lakes of pus (1–3). Studies suggest that GPP flares can be potentially life-threatening due to complications such as sepsis and multisystem organ failure (5, 38, 39). The higher mortality rates associated with GPP compared to other forms of psoriasis underscore the urgent need for treatments that can rapidly control the disease. This study demonstrates that IL-36 inhibitors can achieve a 40% (95% CI 27%-54%) GPPASI75 response rate and a 55% (95% CI 41%-68%) GPPGA (0,1) response rate within 2 weeks, significantly outperforming other biologics.

Additionally, this study reveals that the recurrence rates of GPP within 52 weeks, from highest to lowest, are: IL-36 inhibitors (21% [95% CI 9%-28%]), TNF-alpha inhibitors (20% [95% CI 2%-46%]), IL-17 inhibitors (15% [95% CI 1%-37%]), and IL-23 inhibitors (5% [95% CI 0%-29%]). This suggests that IL-17 inhibitors may offer better long-term flare prevention than IL-36 inhibitors. However, analysis of the long-term preventive effects of IL-36 inhibitors should be interpreted with caution due to the small sample sizes.

In summary, these data demonstrate the significant effectiveness of IL-36 inhibitors in treating GPP, providing rapid control and improvement. Further research is needed to explore the long-term effects on flare prevention.

Furthermore, we observed that 6% (95% CI 1%-11%) of patients experienced severe adverse events, with rates varying among biologics: IL-17 inhibitors (4% [95% CI 0%-14%]), IL-36 inhibitors (5% [95% CI 0%-14%]), IL-23 inhibitors (6% [95% CI 0%-24%]), and TNF-α inhibitors (14% [95% CI 0%-45%]). This suggests that biologics generally have a strong safety profile for the treatment of GPP. IL-36 inhibitors and IL-17 inhibitors demonstrate higher efficacy and better safety profiles compared to IL-23 inhibitors and TNF-α inhibitors in GPP.

This study should be interpreted with several limitations. Moderate heterogeneity and publication bias in the included studies may affect the reliability of the results. The rarity of GPP and the lack of large-scale clinical trials present challenges for deriving evidence-based therapeutic options. Most clinical trials originate from Japan, possibly due to the variation in prevalence across ethnicities, being more common in Asian populations and less so in Caucasian populations (1, 6). Additionally, while GPP primarily affects adults, with a median diagnosis age around 50, it can also occur in children (40). GPP is linked to IL36RN gene mutations (1), which are more frequent in children/adolescents (93.8%) compared to adults (27.5%) (41). Limited data on efficacy and safety in children reduces generalizability to younger populations. Some subgroups include very few studies, decreasing the power of our analysis and the ability to detect effects of certain biologics and long-term flare prevention. These limitations highlight the need for future studies to better address these issues.

## Conclusion

This meta-analysis highlights the efficacy and safety of biologics in patients with GPP, offering evidence for their future clinical application. IL-36 inhibitors deliver a faster and more substantial clinical response compared to other biologics. Further research is needed to assess their role in specific subpopulations and to evaluate their potential in the long-term prevention of flares.
