# Supplementary material for: Biologics for generalized pustular psoriasis: a systematic review and single-arm meta-analysis
Source: Front Immunol. 2024 Oct 14;15:1462158. doi: 10.3389/fimmu.2024.1462158 (PMC11513292; doi:10.3389/fimmu.2024.1462158)
Supplement: Supplementary file 1 [file DataSheet1.docx]

Supplementary Material

# Supplementary Data

# Supplementary Figures and Tables

## Supplementary Figures

**Figure S1. Funnel plot**


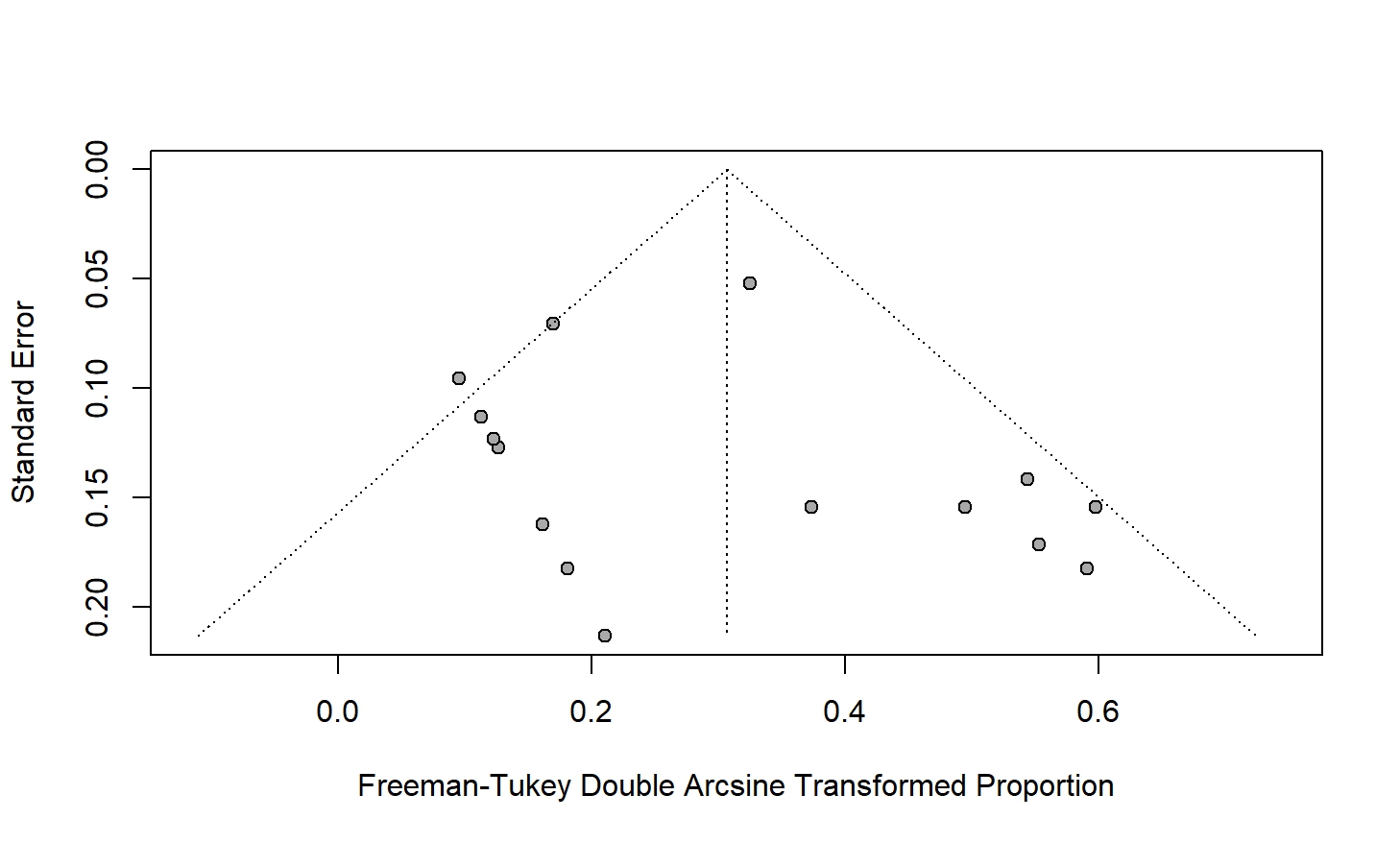


**Figure S2. Sensitivity analysis**

GPPASI 75

2 w


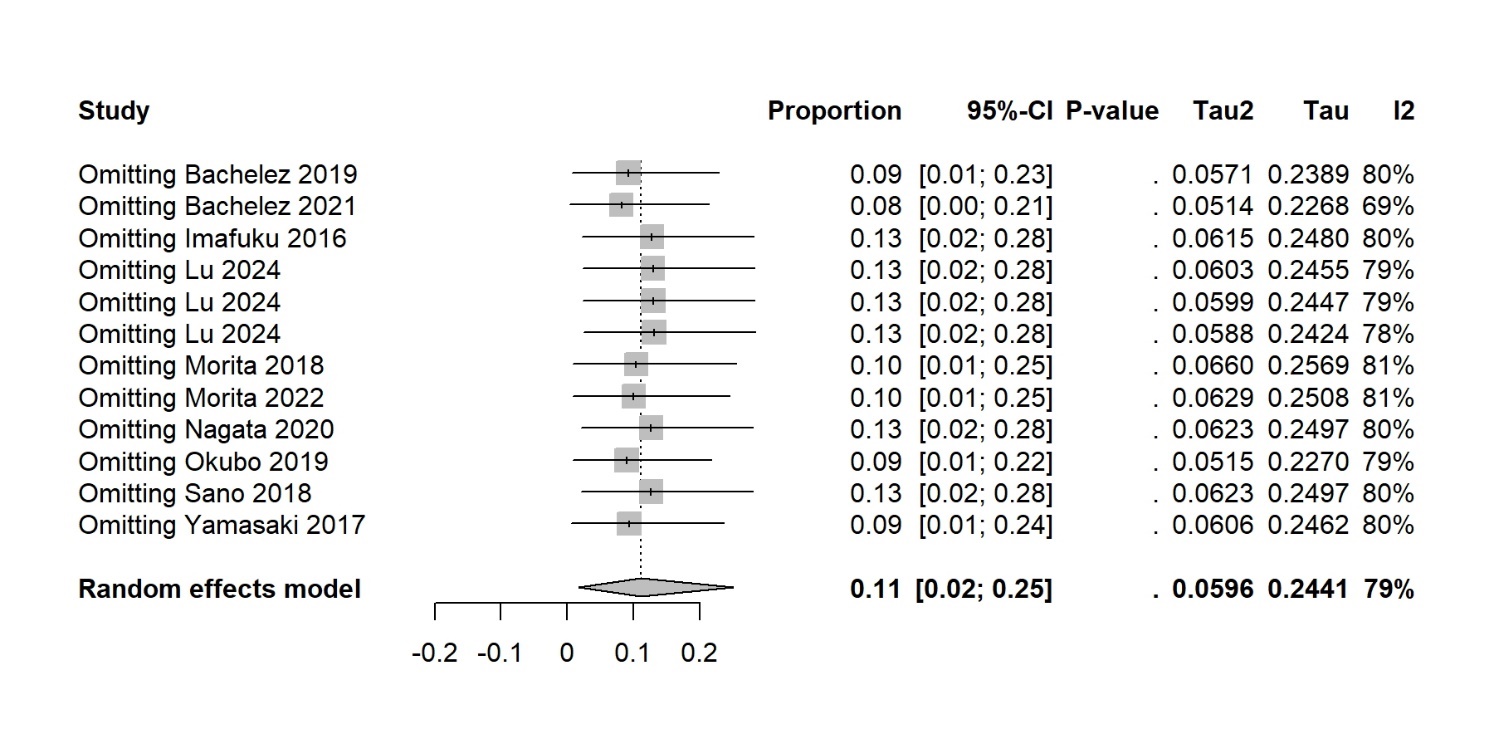


4 w


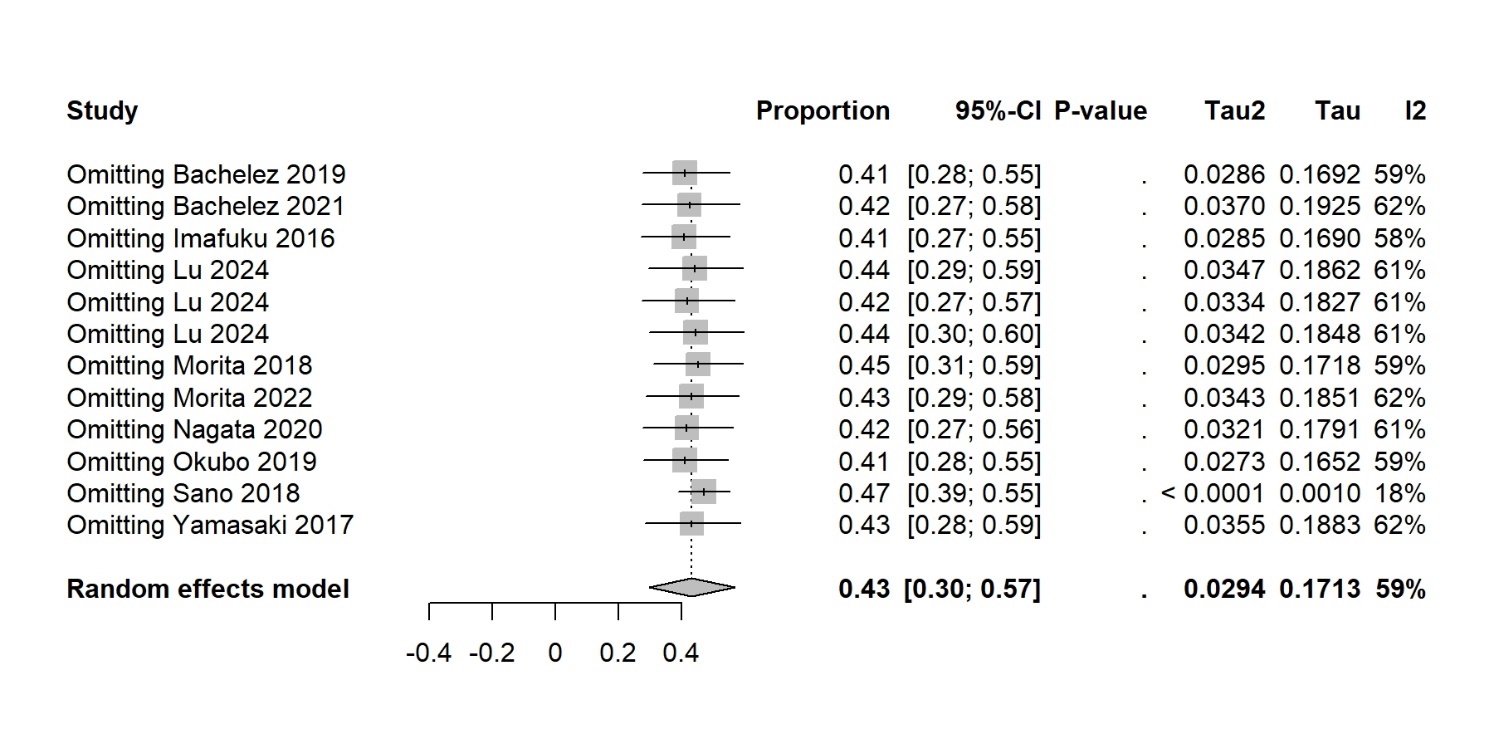


8 w


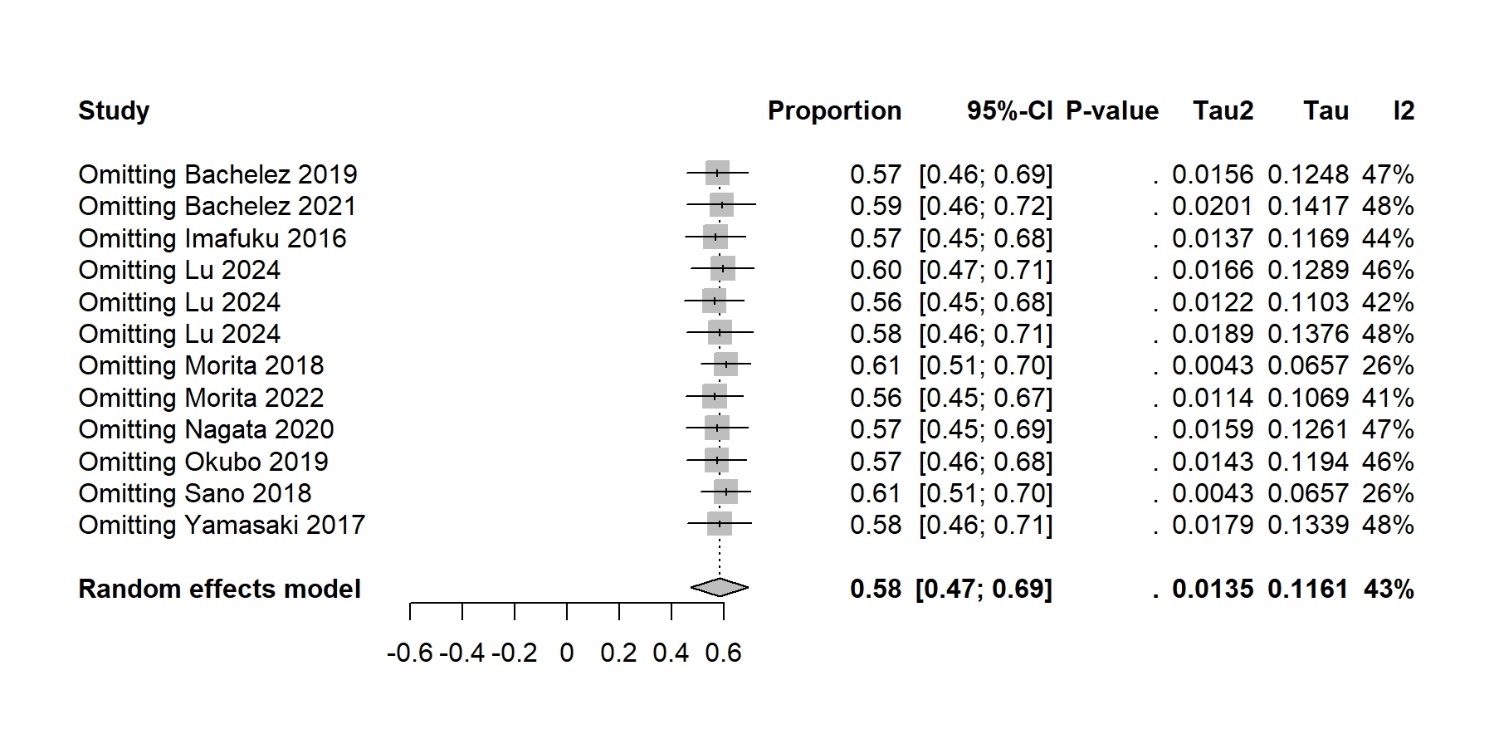


12 w


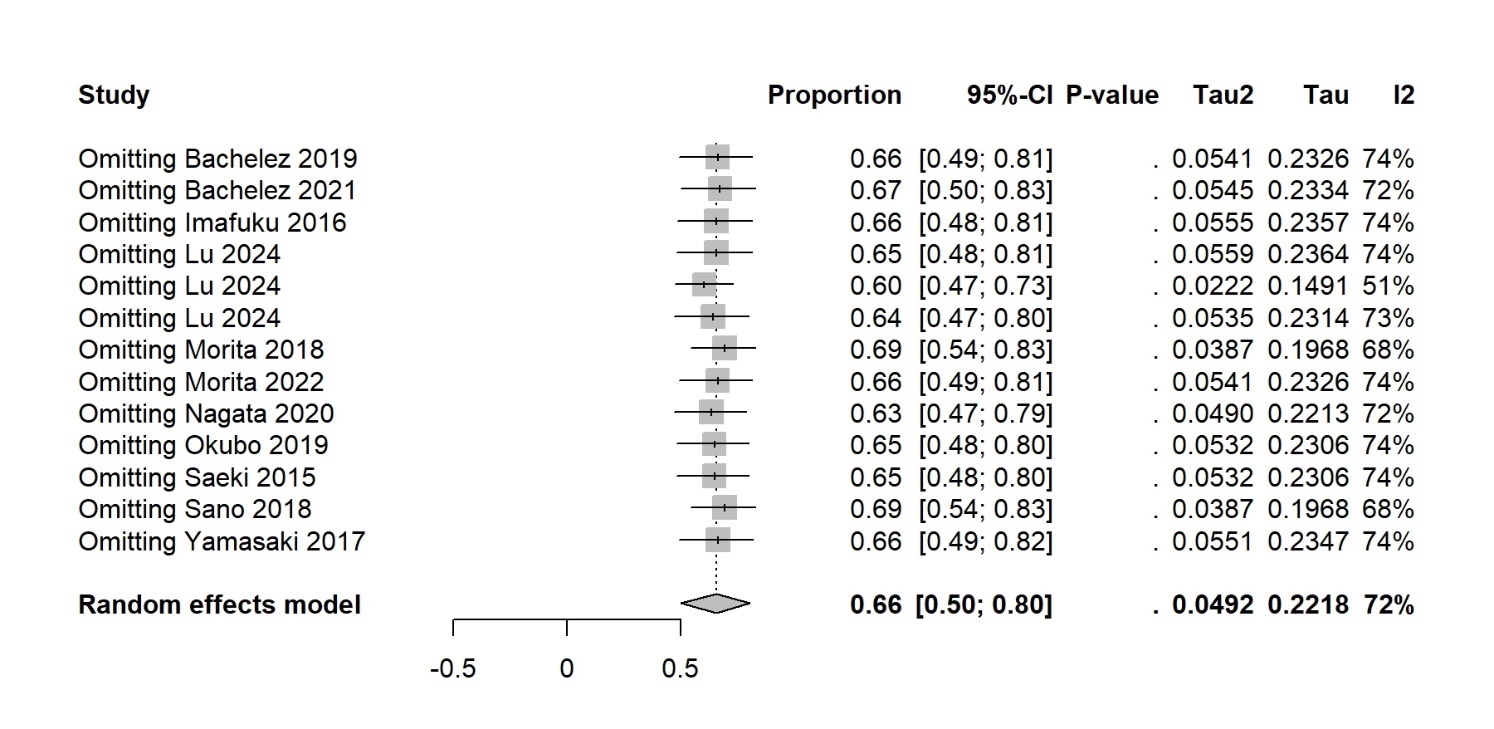


GPPGA (0, 1)

2 w


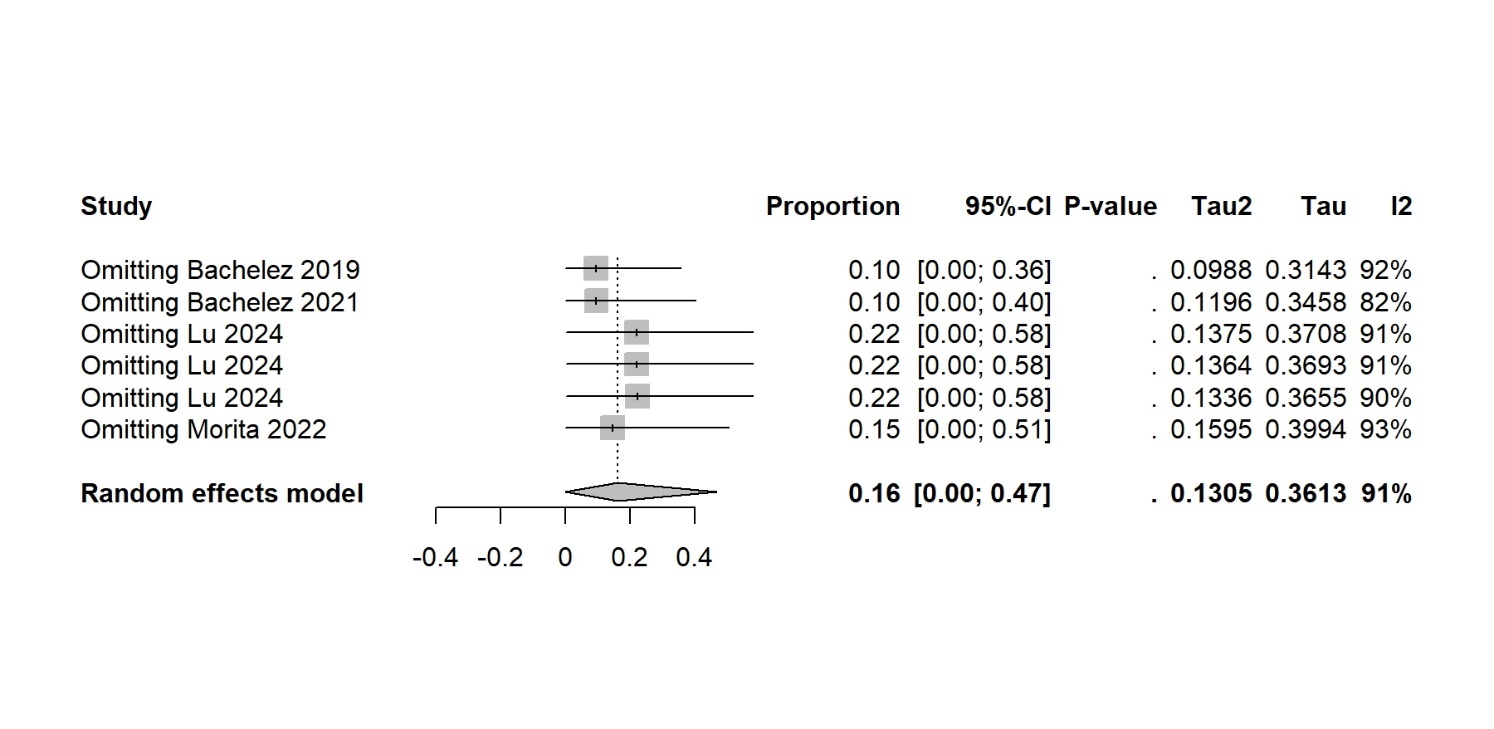


4 w


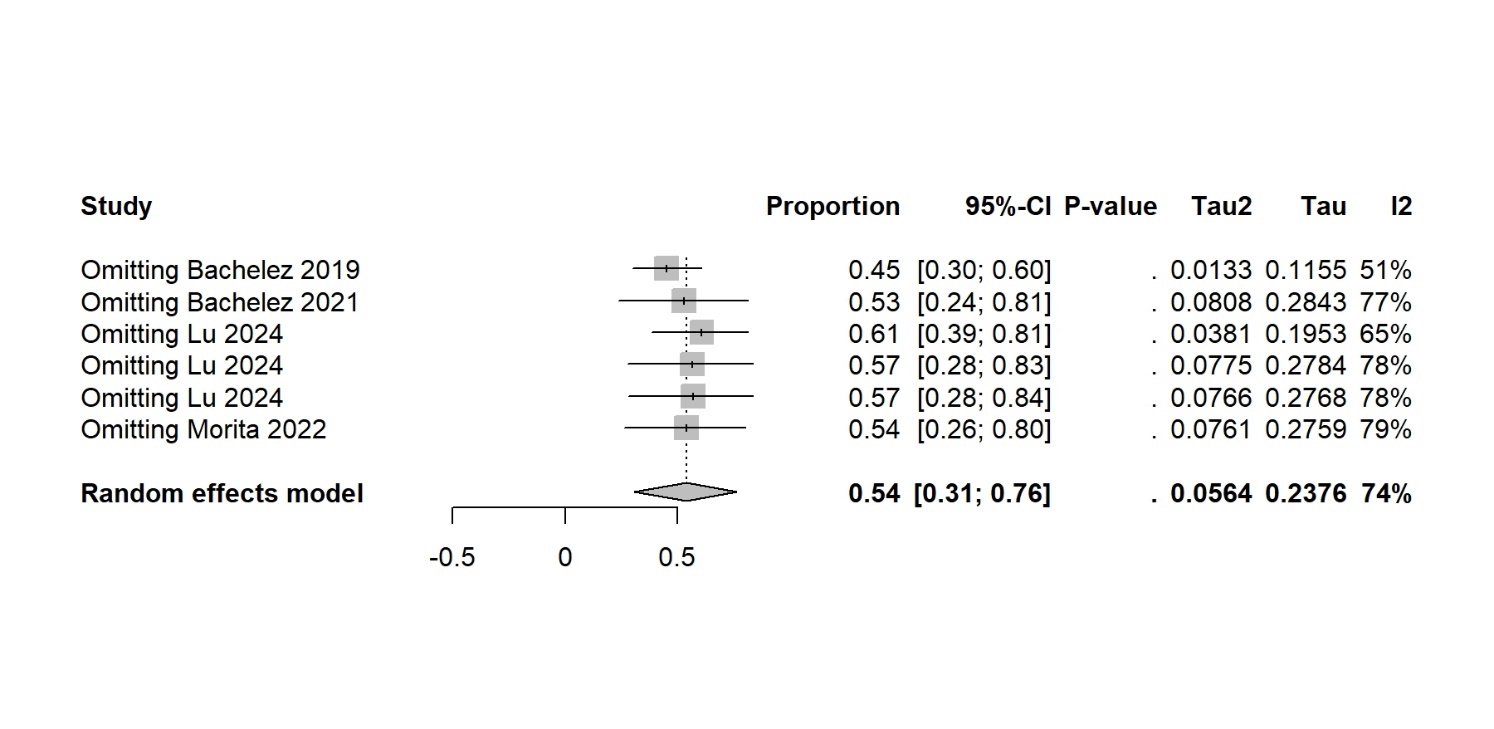


8 w


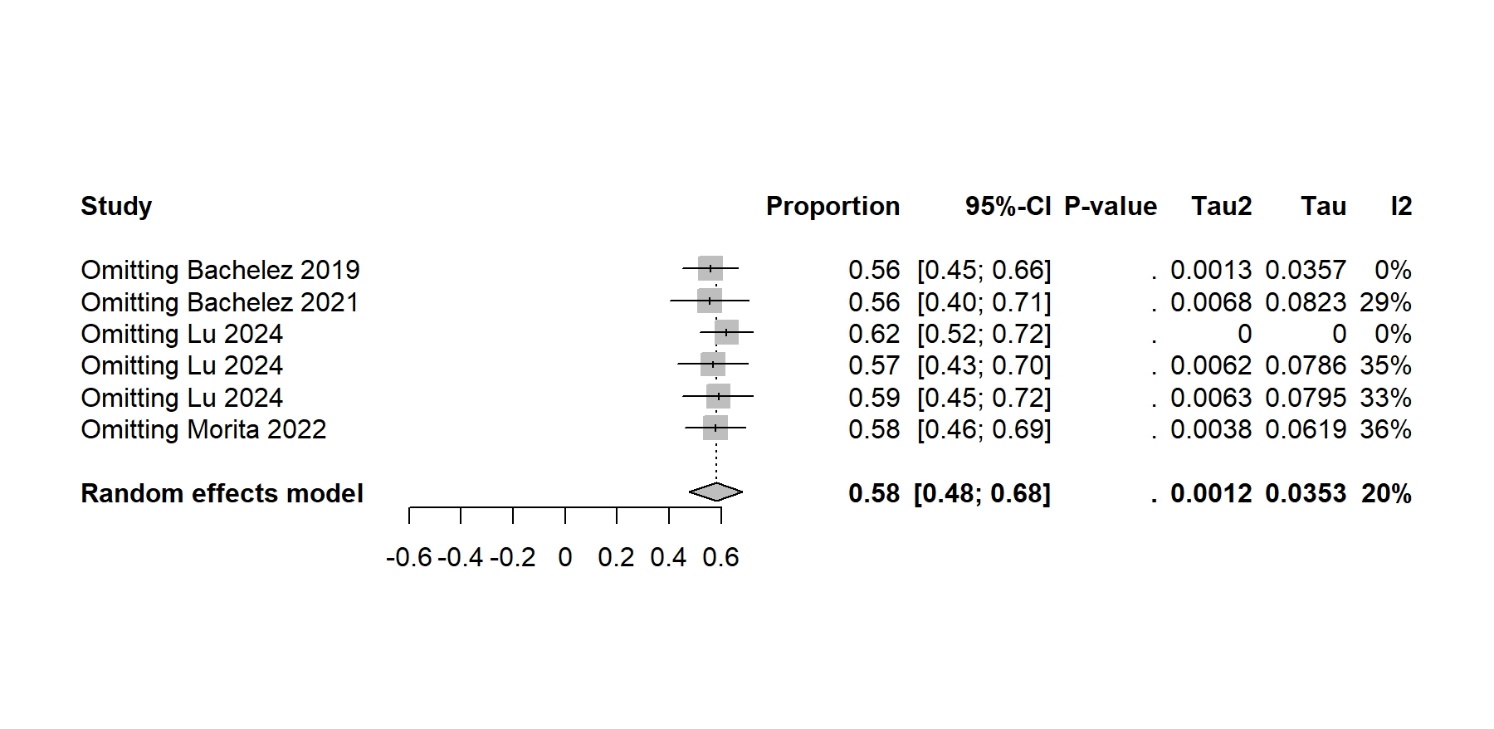


12 w


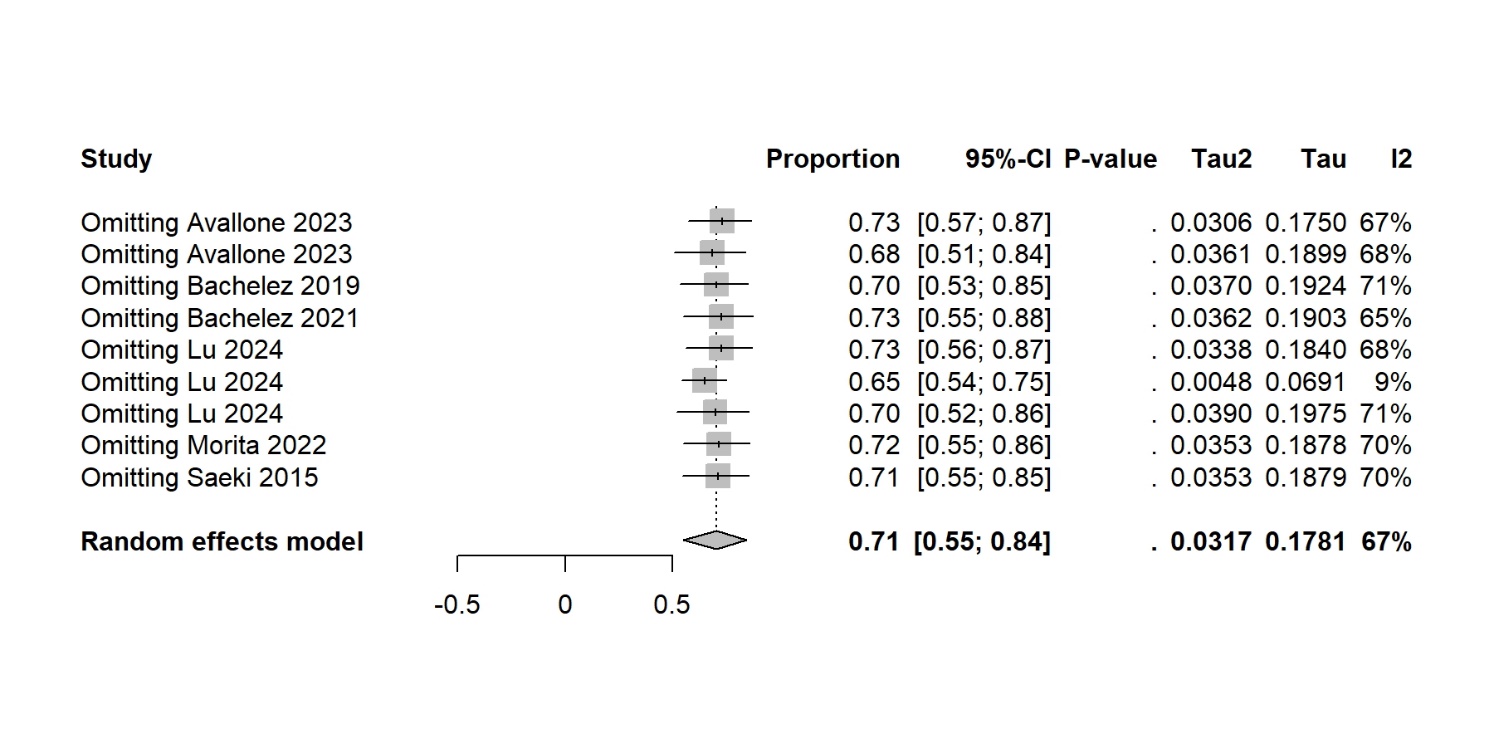


GPP flare


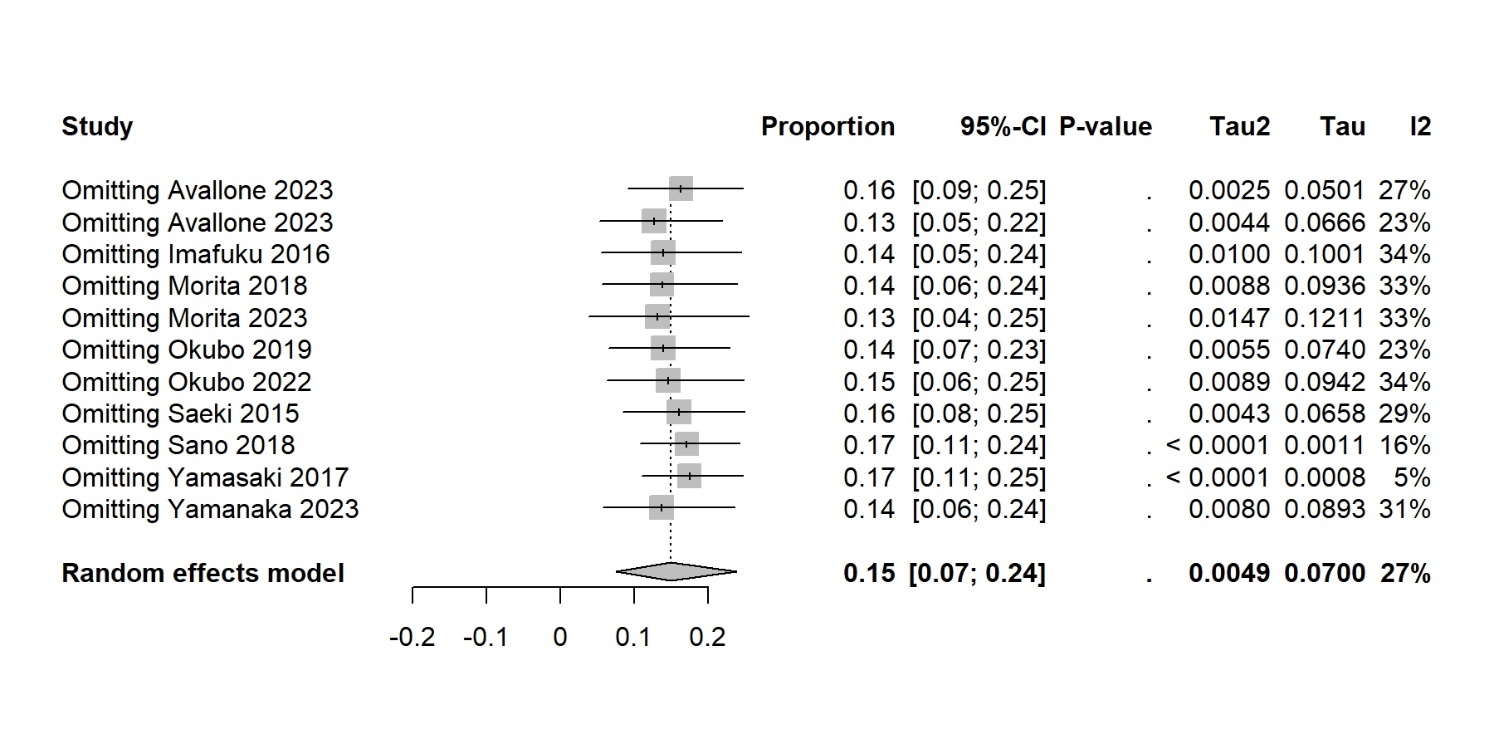


Adverse event


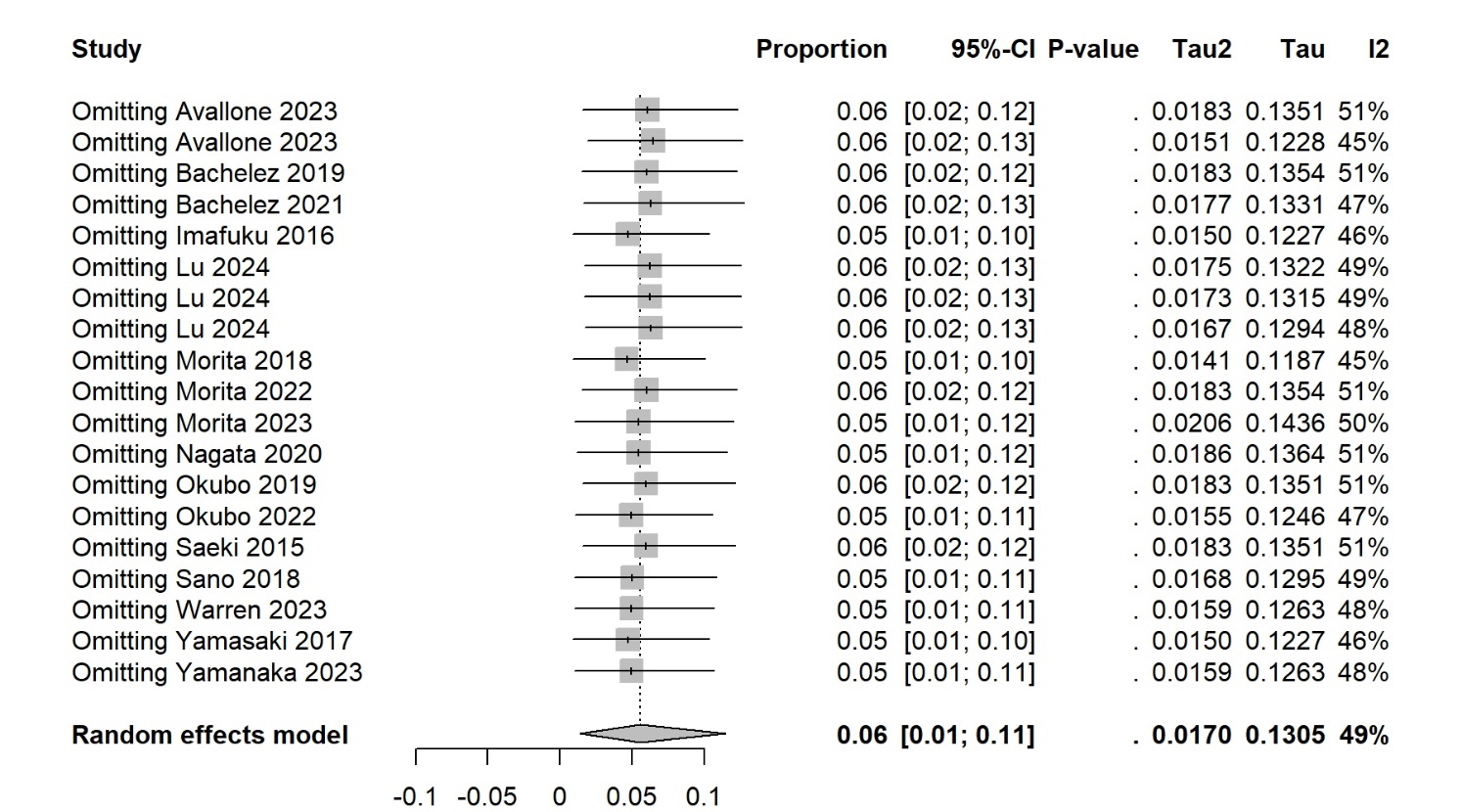


## Supplementary Tables

**Table S1. Search strategy**

| **Pubmed** | | |
| --- | --- | --- |
| 1 | pustular psoriasis[mesh] or pustular psoriasis*[tiab] | 2210 |
| 2 | generalized pustular psoriasis[mesh] or generalized pustular psoriasis*[tiab] | 1088 |
| 3 | GPP*[tiab] | 3224 |
| 4 | 1 or 2 or 3 | 5096 |
| 5 | biologic[mesh] or biologic*[tiab] | 1795845 |
| 6 | spesolimab[mesh] or spesolimab*[tiab] or BI 655130[tiab] or BI655130[tiab] | 79 |
| 7 | adalimumab[mesh] or adalimumab*[tiab] or humira[tiab] or amjevita[tiab] or cyltezo[tiab] or D2E7 Antibody[tiab] | 11589 |
| 8 | infliximab[mesh] or infliximab*[tiab] or MAb cA2[tiab] or monoclonal Antibody cA2[tiab] or inflectra[tiab] or remicade[tiab] or renflexis[tiab] | 18303 |
| 9 | etanercept[mesh] or etanercept*[tiab] or TNR 001[tiab] or TNT Receptor Fusion Protein[tiab] or TNTR Fc[tiab] or TNR-001[tiab] or TNR001[tiab] or Etanercept-szzs[tiab] or TNF Receptor Type II IgG Fusion Protein[tiab] or erelzi[tiab] or Enbrel[tiab] | 9959 |
| 10 | certolizumab[mesh] or certolizumab*[tiab] or Cimzia[tiab] or CDP870[tiab] or CDP 870[tiab] | 1678 |
| 11 | Brodalumab[mesh] or Brodalumab*[tiab] or Siliq[tiab] or KHK-4827[tiab] or KHK4827[tiab] or AMG-827[tiab] or AMG 827[tiab] | 546 |
| 12 | ixekizumab[mesh] or ixekizumab*[tiab] or Taltz[tiab] or LY2439821[tiab] or LY-2439821[tiab] | 1062 |
| 13 | secukinumab[mesh] or secukinumab*[tiab] or Cosentyx[tiab] or AIN 457[tiab] or AIN457[tiab] | 2097 |
| 14 | ustekinumab[mesh] or ustekinumab*[tiab] or Stelara[tiab] or CNTO 1275[tiab] | 3464 |
| 15 | guselkumab[mesh] or guselkumab*[tiab] or Tremfya[tiab] or CNTO 1959[tiab] | 676 |
| 16 | risankizumab[mesh] or risankizumab*[tiab] or ABBV-066[tiab] or skyrizi[tiab] or BI 655066[tiab] | 480 |
| 17 | Tildrakizumab[mesh] or tildrakizumab*[tiab] or SCH 900222[tiab] or Ilumya[tiab] or MK-3222[tiab] | 293 |
| 18 | tocilizumab[mesh] or tocilizumab*[tiab] or atlizumab[tiab] or BAT-1806[tiab] or BAT1806[tiab] or MSB11456[tiab] or MSB-11456[tiab] or RG-1569[tiab] or R-1569[tiab] or RO-4877533[tiab] or Actemra[tiab] or Roactemra[tiab] or RHPM-1[tiab] | 7094 |
| 19 | rituximab[mesh] or rituximab*[tiab] or Mabthera[tiab] or IDEC C2B8[tiab] or GP2013[tiab] or Rituxan[tiab] | 32351 |
| 20 | bimekizumab[mesh] or bimekizumab*[tiab] or UCB4940[tiab] or UCB-4940[tiab] | 181 |
| 21 | golimumab[mesh] or golimumab*[tiab] or CNTO 148[tiab] or Simponi[tiab] | 1535 |
| 22 | anakinra[mesh] or anakinra*[tiab] or Interleukin 1 Receptor Antagonist Protein[tiab] or IL1 Febrile Inhibitor[tiab] or IL-1Ra[tiab] or Urine Derived IL1 Inhibitor[tiab] or Urine IL-1 Inhibitor[tiab] or Antril[tiab] or Kineret[tiab] | 11174 |
| 23 | canakinumab[mesh] or canakinumab*[tiab] or Ilaris[tiab] or ACZ-885[tiab] or ACZ885[tiab] | 1007 |
| 24 | gevokizumab[mesh] or gevokizumab*[tiab] or XMA 005.2[tiab] or XMA005.2[tiab] or XOMA 052[tiab] or XOMA052[tiab] | 61 |
| 25 | acitretin[mesh] or acitretin*[tiab] or Etretin[tiab] or Isoacitretin[tiab] or Isoetretin[tiab] or Ro 10-1670[tiab] or Ro 101670[tiab] or Ro-10-1670[tiab] or Ro101670[tiab] or 13-cis-Acitretin[tiab] or Neotigason[tiab] or Soriatane[tiab] or Ro-13-7652[tiab] or Ro137652[tiab] or Ro 13-7652[tiab] or Ro 137652[tiab] | 2174 |
| 26 | Methotrexate[mesh] or Methotrexate*[tiab] or Amethopterin[tiab] or Mexate[tiab] | 62085 |
| 27 | ciclosporin[mesh] or ciclosporin*[tiab] or Cyclosporin[tiab] or Neoral[tiab] or CyA NOF[tiab] or Sandimmun*[tiab] or CsA Neoral[tiab] or CsANeoral[tiab] or OL 27 400[tiab] or OL 27400[tiab] | 43228 |
| 28 | 5 or 6 or 7or 8 or 9 or 10 or 11 or 12 or 13 or 14 or 15 or 16 or 17 or 18 or 19 or 20 or 21 or 22 or 23 or 24 or 25 or 26 or 27 | 1953243 |
| 29 | trial[mesh] or trial*[tiab] or clinical stud*[tiab] or stud*[tiab] | 14794440 |
| 30 | #4 and #28 and #29 | 239 |
| **Embase** | | |
| 1 | ‘pustular psoriasis’/exp or ‘pustular psoriasis’:ab,ti or ‘pustulosis palmoplantaris’:ab,ti or ‘impetigo herpetiformis’:ab,ti | 6739 |
| 2 | ‘generalized pustular psoriasis’/exp or ‘generalized pustular psoriasis’:ab,ti | 1598 |
| 3 | ‘GPP’:ab,ti | 3734 |
| 4 | 1 or 2 or 3 | 9872 |
| 5 | ‘biologic’/exp or ‘biologic’:ab,ti or ‘spesolimab’/exp or ‘spesolimab’:ab,ti or ‘adalimumab’/exp or ‘adalimumab’:ab,ti or ‘infliximab’/exp or ‘infliximab’:ab,ti or ‘etanercept’/exp or ‘etanercept’:ab,ti or ‘certolizumab’/exp or ‘certolizumab’:ab,ti or ‘Brodalumab’/exp or ‘Brodalumab’:ab,ti or ‘ixekizumab’/exp or ‘ixekizumab’:ab,ti or ‘secukinumab’/exp or ‘secukinumab’:ab,ti or ‘ustekinumab’/exp or ‘ustekinumab’:ab,ti or ‘guselkumab’/exp or ‘guselkumab’:ab,ti or ‘risankizumab’/exp or ‘risankizumab’:ab,ti or ‘Tildrakizumab’/exp or ‘tildrakizumab’:ab,ti or ‘tocilizumab’/exp or ‘tocilizumab’:ab,ti or ‘rituximab’/exp or ‘rituximab’:ab,ti or ‘bimekizumab’/exp or ‘bimekizumab’:ab,ti or ‘golimumab’/exp or ‘golimumab’:ab,ti or ‘Anakinra’/exp or ‘anakinra’:ab,ti or ‘canakinumab’/exp or ‘canakinumab’:ab,ti or ‘gevokizumab’/exp or ‘gevokizumab’:ab,ti or ‘acitretin’/exp or ‘acitretin’:ab,ti or ‘isoetretin’:ab,ti or ‘Methotrexate’/exp or ‘Methotrexate’:ab,ti or ‘ciclosporin’/exp or ‘ciclosporin’:ab,ti | 1557121 |
| 6 | ‘trial’/exp or ‘trial’:ab,ti or ‘clinical study’:ab,ti or ‘clinical studies’:ab,ti or ‘study’:ab,ti or ‘studies’:ab,ti | 17844881 |
| 7 | [article]/lim or [article in press]/lim or [review]/lim |  |
| 8 | 4 and 5 and 6 and 7 | 349 |
| **Cochrane library** | | |
| 1 | (pustular psoriasis or pustular psoriasis*):ti,ab,kw | 166 |
| 2 | (generalized pustular psoriasis or generalized pustular psoriasis*):ti,ab,kw | 74 |
| 3 | (GPP*):ti,ab,kw | 115 |
| 4 | #1 or #2 or #3 | 237 |
| 5 | (biologic or biologic*):ti,ab,kw | 42828 |
| 6 | (spesolimab or spesolimab* or BI 655130 or BI655130):ti,ab,kw | 62 |
| 7 | (adalimumab or adalimumab* or humira or amjevita or cyltezo or D2E7 Antibody):ti,ab,kw | 4008 |
| 8 | (infliximab or infliximab* or MAb cA2 or monoclonal Antibody cA2 or inflectra or remicade or renflexis):ti,ab,kw | 2726 |
| 9 | (etanercept or etanercept* or TNR 001 or TNT Receptor Fusion Protein or TNTR Fc or TNR-001 or TNR001 or Etanercept-szzs or TNF Receptor Type II IgG Fusion Protein or erelzi or Enbrel):ti,ab,kw | 2460 |
| 10 | (certolizumab or certolizumab* or Cimzia or CDP870 or CDP 870):ti,ab,kw | 787 |
| 11 | (Brodalumab or Brodalumab* or Siliq or KHK-4827 or KHK4827 or AMG-827 or AMG 827):ti,ab,kw | 208 |
| 12 | (ixekizumab or ixekizumab* or Taltz or LY2439821 or LY-2439821):ti,ab,kw | 671 |
| 13 | (secukinumab or secukinumab* or Cosentyx or AIN 457 or AIN457):ti,ab,kw | 1175 |
| 14 | (ustekinumab or ustekinumab* or Stelara or CNTO 1275):ti,ab,kw | 1199 |
| 15 | (guselkumab or guselkumab* or Tremfya or CNTO 1959):ti,ab,kw | 629 |
| 16 | (risankizumab or risankizumab* or ABBV-066 or skyrizi or BI 655066):ti,ab,kw | 309 |
| 17 | (Tildrakizumab or tildrakizumab* or SCH 900222 or Ilumya or MK-3222):ti,ab,kw | 207 |
| 18 | (tocilizumab or tocilizumab* or atlizumab or BAT-1806 or BAT1806 or MSB11456 or MSB-11456 or RG-1569 or R-1569 or RO-4877533 or Actemra or Roactemra or RHPM-1):ti,ab,kw | 1694 |
| 19 | (rituximab or rituximab* or Mabthera or IDEC C2B8 or GP2013 or Rituxan):ti,ab,kw | 6128 |
| 20 | (bimekizumab or bimekizumab* or UCB4940 or UCB-4940):ti,ab,kw | 247 |
| 21 | (golimumab or golimumab* or CNTO 148 or Simponi):ti,ab,kw | 802 |
| 22 | (anakinra or anakinra* or Interleukin 1 Receptor Antagonist Protein or IL1 Febrile Inhibitor or IL-1Ra or Urine Derived IL1 Inhibitor or Urine IL-1 Inhibitor or Antril or Kineret):ti,ab,kw | 1399 |
| 23 | (canakinumab or canakinumab* or Ilaris or ACZ-885 or ACZ885):ti,ab,kw | 399 |
| 24 | (gevokizumab or gevokizumab* or XMA 005.2 or XMA005.2 or XOMA 052 or XOMA052):ti,ab,kw | 33 |
| 25 | (acitretin or acitretin* or Etretin or Isoacitretin or Isoetretin or Neotigason or Soriatane):ti,ab,kw | 217 |
| 26 | (Methotrexate or Methotrexate* or Amethopterin or Mexate):ti,ab,kw | 13047 |
| 27 | (ciclosporin or ciclosporin* or Cyclosporin or Neoral or CyA NOF or Sandimmun* or CsA Neoral or CsANeoral or OL 27 400 or OL 27400):ti,ab,kw | 3379 |
| 28 | #5 or #6 or #7 or #8 or #9 or #10 or #11 or #12 or #13 or #14 or #15 or #16 or #17 or #18 or #19 or #20 or #21 or #22 or #23 or #24 or #25 or #26 or #27 | 72167 |
| 29 | (trial or trial* or random* or clinical stud* or stud*):ti,ab,kw | 1855393 |
| 30 | #4 and #28 and #29 | 100 |
| Pubmed + Embase + Cochrane library = 688 | | |
| 688 - duplicates =688 - 149= 539 | | |

**Table S2. GRADE assessment**

| **Certainty assessment** | | | | | | | **No. of participants** | **Effect** | **Certainty** | **Importance** |
| --- | --- | --- | --- | --- | --- | --- | --- | --- | --- | --- |
| **No. of studies** | **Study Design** | **Risk of Bias** | **Inconsistency** | **Indirectness** | **Imprecision** | **Others** | **Intervention** | **Relative (95% CI)** |  |  |
| GPPASI 75 (2 w) | | | | | | | | | |  |
| 10 | Randomized trial and observational study | Very serious ^a^ | No | No | Serious ^b^ | No | 34/173 (19.7%) | **Proportion 0.11** (0.02 to 0.25) | ⨁◯◯◯ Very low | 9 |
| GPPASI 75 (4 w) | | | | | | | | | | |
| 10 | Randomized trial and observational study | Very serious ^a^ | No | No | Serious ^b^ | No | 77/173 (44.5%) | **Proportion 0.43** (0.30 to 0.57) | ⨁◯◯◯ Very low | 9 |
| GPPASI 75 (8 w) | | | | | | | | | |  |
| 10 | Randomized trial and observational study | Very serious ^a^ | No | No | Serious ^b^ | No | 99/173 (57.2%) | **Proportion 0.58** (0.47 to 0.69) | ⨁◯◯◯ Very low | 9 |
| GPPASI 75 (12 w) | | | | | | | | | |  |
| 11 | Randomized trial and observational study | Very Serious ^a^ | No | No | Serious ^b^ | No | 111/178 (62.4%) | **Proportion 0.66** (0.50 to 0.80) | ⨁◯◯◯ Very low | 9 |
| GPPGA (0, 1) (2 w) | | | | | | | | | |  |
| 5 | Randomized trial and observational study | Serious ^c^ | No | No | Serious ^b^ | No | 37/124 (29.8%) | **Proportion 0.19** (0.01 to 0.46) | ⨁⨁◯◯ Low | 9 |
| GPPGA (0, 1) (4 w) | | | | | | | | | |  |
| 5 | Randomized trial and observational study | Serious ^c^ | No | No | Serious ^b^ | No | 67/124 (54.0%) | **Proportion 0.58** (0.36 to 0.78) | ⨁⨁◯◯ Low | 9 |
| GPPGA (0, 1) (8 w) | | | | | | | | | |  |
| 5 | Randomized trial and observational study | Serious ^c^ | No | No | Serious ^b^ | No | 74/124 (59.7%) | **Proportion 0.60** (0.50 to 0.70) | ⨁⨁◯◯ Low | 9 |
| GPPGA (0, 1) (12 w) | | | | | | | | | |  |
| 7 | Randomized trial and observational study | Serious ^c^ | No | No | Serious ^b^ | No | 43/62 (69.4%) | **Proportion 0.70** (0.56 to 0.82) | ⨁⨁◯◯ Low | 9 |
| GPP flare | | | | | | | | | |  |
| 10 | Randomized trial and observational study | Very serious ^a^ | No | No | Serious ^b^ | No | 34/174 (19.5%) | **Proportion 0.15** (0.07 to 0.24) | ⨁◯◯◯ Very low | 9 |
| Adverse events | | | | | | | | | |  |
| 16 | Randomized trial and observational study | Very serious ^d^ | No | No | Serious ^b^ | No | 28/329 (8.5%) | **Proportion 0.06** (0.01 to 0.11) | ⨁◯◯◯ Very low | 8 |

**CI:** Confidence interval

#### Explanations

a. Four studies are at high risk of bias.

b. Small sample size.

c. One study is at high risk of bias.

d. Five studies are at high risk of bias.

**Table S3. Summary of excluded studies**

| **Study** | **Title** | **Reason** |
| --- | --- | --- |
| Akaji 2021 | Generalized pustular psoriasis associated with systemic lupus erythematosus successfully treated with secukinumab | Case report |
| Anadkat 2022 | 291 Improvements in GPPGA score in patients experiencing a generalized pustular psoriasis (GPP) flare: effisayil 1 study results | Duplicates ([NCT03782792](http://clinicaltrials.gov/show/NCT03782792)) |
| Arakawa 2016 | Therapeutic Efficacy of Interleukin 12/Interleukin 23 Blockade in Generalized Pustular Psoriasis Regardless of IL36RN Mutation Status | No outcomes of interest reported |
| Babuna 2020 | Infliximab for the treatment of recalcitrant generalized pustular psoriasis of pregnancy: Report of a challenging case | Case report |
| Bachelez 2022 | Efficacy of spesolimab for the rapid control of generalized pustular psoriasis flares: results from the placebo-controlled Effisayil™ 1 study | Duplicates ([NCT03782792](http://clinicaltrials.gov/show/NCT03782792)) |
| Böhner 2016 | Acute Generalized Pustular Psoriasis Treated With the IL-17A Antibody Secukinumab | Case report |
| Brenner 2009 | Generalized pustular psoriasis induced by systemic glucocorticosteroids: four cases and recommendations for treatment | Case report |
| Burden 2023 | Efficacy of spesolimab for the treatment of generalized pustular psoriasis flares across pre-specified patient subgroups in the Effisayil 1 study | Duplicates ([NCT03782792](http://clinicaltrials.gov/show/NCT03782792)) |
| Chandran 2010 | A dramatic response to a single dose of infliximab as rescue therapy in acute generalized pustular psoriasis of von Zumbusch associated with a neutrophilic cholangitis | Case report |
| Coscarella 2023 | Low grade of satisfaction related to the use of current systemic therapies among pustular psoriasis patients: a therapeutic unmet need to be fulfilled | No outcomes of interest reported |
| Elewski 2022 | 32924 Sustained treatment effect of spesolimab over 12 weeks for generalized pustular psoriasis flares; results from the Effisayil 1 study | Duplicates ([NCT03782792](http://clinicaltrials.gov/show/NCT03782792)) |
| Elewski 2023 | Rapid and sustained improvements in Generalized Pustular Psoriasis Physician Global Assessment scores with spesolimab for treatment of generalized pustular psoriasis flares in the randomized, placebo-controlled Effisayil 1 study | Duplicates ([NCT03782792](http://clinicaltrials.gov/show/NCT03782792)) |
| Hsu 2024 | Acquired diffuse palmoplantar erythema with keratoderma in Chinese patients with pustular psoriasis: A predictor for IL36 receptor antagonist c.115+6T>C mutation? | No outcomes of interest reported |
| Li 2022 | 094 Characterisation of the immunogenicity of spesolimab in patients with a generalized pustular psoriasis (GPP) flare | Duplicates ([NCT03782792](http://clinicaltrials.gov/show/NCT03782792)) |
| Miyachi 2022 | Treatments and outcomes of generalized pustular psoriasis: A cohort of 1516 patients in a nationwide inpatient database in Japan | No outcomes of interest reported |
| Morita 2023 | Efficacy and safety of spesolimab in Asian patients with a generalized pustular psoriasis flare: Results from the randomized, double-blind, placebo-controlled Effisayil™ 1 study | Duplicates ([NCT03782792](http://clinicaltrials.gov/show/NCT03782792)) |
| Navarini 2022 | 33005 Clinically significant improvements in patient-reported outcomes (PROs) in patients with a generalized pustular psoriasis (GPP) flare treated with spesolimab | Duplicates ([NCT03782792](http://clinicaltrials.gov/show/NCT03782792)) |
| Navarini 2023 | Spesolimab improves patient-reported outcomes in patients with generalized pustular psoriasis: Results from the Effisayil 1 study | Duplicates ([NCT03782792](http://clinicaltrials.gov/show/NCT03782792)) |
| Saeki 2017 | Efficacy and safety of ixekizumab treatment for Japanese patients with moderate to severe plaque psoriasis, erythrodermic psoriasis and generalized pustular psoriasis: Results from a 52-week, open-label, phase 3 study (UNCOVER-J) | Duplicates ([NCT03782792](http://clinicaltrials.gov/show/NCT03782792)) |
| Tada 2024 | Treatment patterns and drug survival for generalized pustular psoriasis: A patient journey study using a Japanese claims database | No outcomes of interest reported |
| Torres 2024 | Clinical course and disease burden of patients with generalized pustular psoriasis in Portugal: a multicenter retrospective cohort study | Wrong study design |
| Tsai 2022 | 379 Efficacy of spesolimab in patients with generalized pustular psoriasis (GPP) flares with and without systemic inflammation | Duplicates ([NCT03782792](http://clinicaltrials.gov/show/NCT03782792)) |
| Tsai 2023 | Efficacy and Safety of Spesolimab in Patients with Generalized Pustular Psoriasis: A Subgroup Analysis of Chinese Patients in the Effisayil 1 Trial | Duplicates ([NCT03782792](http://clinicaltrials.gov/show/NCT03782792)) |
| van de Kerkhof 2022 | 104 The effect of present or historical psoriasis on the efficacy of spesolimab in patients with a generalized pustular psoriasis (GPP) flare | Duplicates ([NCT03782792](http://clinicaltrials.gov/show/NCT03782792)) |
| Warren 2023 | 43754 Efficacy and safety of spesolimab for the treatment of generalized pustular psoriasis flares in hospitalized versus non-hospitalized patients | Duplicates ([NCT03782792](http://clinicaltrials.gov/show/NCT03782792)) |

**Table S4. Adverse Events**

| **Study** | **Drug** | **Target** | **Duration** | **Region** | **Race** | **Adverse events*** |
| --- | --- | --- | --- | --- | --- | --- |
| Avallone 2023 | NR | IL-23  IL-17 | NR | Italy | White: 36 | Asthenia (1), pustulation on the back (1), hand/foot pain after first administration (1), increased hunger in the days following the injection (1), injection site reaction (1) |
| Bachelez 2019 | Spesolimab | IL-36 | Once | Tunisia, France, Malaysia, Korea, Taiwan | Asian: 4  White: 2  N/A: 1 | Eosinophilia (2), vomiting (1), chills (1), pain (1), upper respiratory tract infection (2), urinary tract infection (1), infusion-related reaction (1), arthralgia (1) |
| Bachelez 2021 | Spesolimab | IL-36 | Once | China, France, Germany, Japan, Korea, Malaysia, Singapore, Switzerland, Taiwan, Thailand, Tunisia, United States | White = 19  Asian = 16 | Pyrexia (2) |
| Imafuku 2016 | Secukinumab | IL-17 | 52 w | Japan | Asian: 12 | Nasopharyngitis (6), urticaria (2), diabetes mellitus (2), arthralgia (2), Bowen's disease and cellulitis (1), drug-induced liver injury (1), upper gastrointestinal hemorrhage, hypoglycemia and abnormal hepatic function (1) |
| Lu 2024 | Adalimumab/ Guselkumab/ Secukinumab | TNF-α/ IL-23/ IL-17 | 12 w | China | Asian: 50 | Red and swollen (4), injection site pain (9), upper respiratory tract infection (4), dry mucosa (40), peeling hands and feet (32), dyslipidemia (16), COIVD-19 infection (37) |
| Morita 2018 | Adalimumab | TNF-α | 52 w | Japan | Asian: 10 | Infection (2), nasopharyngitis (1), upper respiratory tract infection (1), dermatitis contact (1), upper respiratory tract infection (1) |
| Morita 2022 | Ixekizumab | IL-17 | 12 w | Japan | Asian: 7 | Infection (7), worsening or new onset of psoriasis (2), liver failure or other liver event (1), nasopharyngitis (3), pruritus (3), hypoalbuminemia (2), serious infection (2), congestive heart failure (1) |
| Morita 2023 | Spesolimab | IL-36 | 48 w | Argentina, Belgium, Chile, China, France, Germany, Italy, Japan, Malaysia, Mexico, Philippines, Republic of Korea, Russia, Spain, Taiwan, Thailand, Tunisia, Turkey, USA, Vietnam | White = 30  Asian = 62 | Skin and subcutaneous tissue disorders (50), infections and infestations (31), general disorders and administration site conditions (25), musculoskeletal and connective tissue disorders (13) |
| Nagata 2020 | Ixekizumab | IL-17 | NR | Japan | Asian: 10 | None |
| Okubo 2019 | Ixekizumab | IL-17 | 52 w | Japan | Asian: 5 | Infection (4), allergic reaction/hypersensitivity (2), injection site reaction (1) |
| Okubo 2022 | Certolizumab | TNF-α | 52 w | Japan | Asian: 7 | Nasopharyngitis (2), pruritus (1), worsening or new onset of psoriasis (1), neutropenia (1), pustular psoriasis (1) |
| Saeki 2015 | Ixekizumab | IL-17 | 52 w | Japan | Asian: 5 | Infection (4), allergic reaction/hypersensitivity (2), injection site reaction (2) |
| Sano 2018 | Guselkumab | IL-23 | 52 w | Japan | Asian: 10 | Infection (5), nasopharyngitis (2), gastroenteritis (1), nausea and vomiting (1), arthralgia (1), alopecia (2), squamous cell carcinoma (1), loss of consciousness (1) |
| Warren 2023 | Imsidolimab | IL-36 | 12 w | UK, Poland | White = 7  Asian = 1 | Blood and lymphatic system disorders (2), anaemia (1), lymphadenopathy (1), gastrointestinal disorders (2), nausea (1), toothache (1), vomiting (1), infections and infestations (2), COVID-19 (1), nosocomial infection (1), respiratory (2), thoracic and mediastinal disorders (2), oropharyngeal pain (2), skin and subcutaneous tissue disorders (2), psoriasis (1), skin haemorrhage (1) |
| Yamasaki 2017 | Brodalumab | IL-17 | 52 w | Japan | Asian: 12 | Liver failure or other liver event (3), scirrhous gastric cancer (1), alcoholic liver disorder (1), others (5) |
| Yamanaka 2023 | Risankizumab | IL-23 | 160 w | Japan | Asian: 8 | Nasopharyngitis (4), diarrhea (2), folliculitis (2), skin papilloma (2), dry skin (2), periarthritis (1), neutrophil count decreased (1), candidiasis (1), injection site reaction (3), lumbar vertebral fracture (1), exacerbation of pustular psoriasis (1), hepatocellular carcinoma (1) |

*Adverse events were coded with the use of the Medical Dictionary for Drug Regulatory Activities, version 20.1. The intensity of adverse events were categorized as mild, moderate, or severe by investigators as per the following criteria: Mild; awareness of sign(s) or symptom(s) that is/are easily tolerated, Moderate; enough discomfort to cause interference with usual activity, Severe; incapacitating or causing inability to work or to perform usual activities.
